# The incidence of opportunistic infections in patients with psoriatic arthritis treated with biologic and targeted synthetic agents: A systematic review and meta-analysis

**DOI:** 10.3389/fphar.2022.992713

**Published:** 2022-10-05

**Authors:** Athanasios Vassilopoulos, Fadi Shehadeh, Gregorio Benitez, Markos Kalligeros, Joanne S. Cunha, Cheston B. Cunha, Eleftherios Mylonakis

**Affiliations:** ^1^ Infectious Diseases Division, Rhode Island Hospital, Providence, RI, United States; ^2^ Warren Alpert Medical School of Brown University, Providence, RI, United States; ^3^ School of Electrical and Computer Engineering, National Technical University of Athens, Athens, Greece

**Keywords:** psoriatic arthritis, opportunistic infections, BDMARDs, tsDMARDs, JAK inhibitors, herpes zoster, *Candida* spp

## Abstract

**Background:** Biologic (bDMARD) and targeted synthetic (tsDMARD) disease-modifying anti-rheumatic drugs have broadened the treatment options and are increasingly used for patients with psoriatic arthritis (PsA). These agents block different pro-inflammatory cytokines or specific intracellular signaling pathways that promote inflammation and can place patients at risk of serious infections. We aimed to review the incidence of opportunistic infections (OIs) in patients with PsA who were treated with these agents.

**Methods:** We searched PubMed and EMBASE through 14 April 2022 for randomized clinical trials evaluating bDMARD or tsDMARD in the treatment of PsA. Trials were eligible if they compared the effect of a bDMARD or tsDMARD with placebo and provided safety data. We used the Revised Cochrane risk-of-bias tool to assess the risk of bias among trials, and stratified the studies by mechanism of action (MOA) of the agents studied.

**Results:** We included 47 studies in this analysis. A total of 17,197 patients received at least one dose of an agent of interest. The cumulative incidence of OIs by MOA was as follows: 1) JAK inhibitors: 2.72% (95% CI: 1.05%–5.04%), 2) anti-IL-17: 1.18% (95% CI: 0.60%–1.9%), 3) anti-IL-23: 0.24% (95% CI: 0.04%–0.54%), and 4) anti-TNFs: 0.01% (95% CI: 0.00%–0.21%). Based on their MOA, these agents are known to increase the risk of certain serious infections. The cumulative incidence of herpes zoster infection following treatment with JAK inhibitors (JAKi) was 2.53% (95% CI: 1.03%–4.57%) and the cumulative incidence of opportunistic *Candida* spp. infections following treatment with anti-IL-17, was 0.97% (95% CI: 0.51%–1.56%).

**Conclusion:** The overall incidence of OIs among patients with PsA who were treated with biologic and targeted synthetic agents is low. However, careful monitoring is warranted for specific OIs such as herpes zoster infection following JAKi treatment, mucocutaneous candidiasis following anti-IL-17 treatment, and *Mycobacterium tuberculosis* infection following anti-TNF treatment.

## 1 Introduction

Novel treatment options for psoriatic arthritis (PsA) significantly decrease disease activity, prevent structural damage, and improve patient quality of life (Husni, 2015; Ogdie et al., 2015; Ogdie et al., 2020; Ruyssen-Witrand et al., 2020; Gupta et al., 2021). These treatments include biologic disease-modifying anti-rheumatic drugs (bDMARDs) and the most recently available oral targeted synthetic DMARDs (tsDMARDs) (Ritchlin et al., 2017; Van den Bosch and Coates, 2018; Singh et al., 2019; Ogdie et al., 2020). bDMARDs target pro-inflammatory cytokines such as tumor necrosis factor (TNF), interleukin 12 (IL-12), interleukin 17 (IL-17), and interleukin 23 (IL-23) as well as T cell activation, that are associated with the pathogenesis of PsA (Ritchlin et al., 2017; Veale and Fearon, 2018; Schett et al., 2021). Furthermore, tsDMARDs suppress intracellular signaling pathways that promote inflammation by inhibiting phosphodiesterase 4 (PDE4) or Janus family kinases (JAK) (Haikarainen et al., 2019).

Serious infections, particularly OIs, are a concern in patients with PsA (Minozzi et al., 2016; Ritchlin et al., 2019; Li et al., 2020). Psoriasis and PsA may increase the risk of infections *via* loss of skin barrier integrity and innate or adaptive immune alterations, while biologic agent use may increase the risk of infections as seen in patients with psoriasis and rheumatoid arthritis (RA) (Bergboer et al., 2012; Singh et al., 2015; Minozzi et al., 2016; Yiu et al., 2016; Subesinghe et al., 2018). Since biologic agents are also used in PsA, they could increase the risk of both serious and OIs.

Given the limited data regarding the risk of OIs in patients with PsA treated with bDMARDs or tsDMARDs, we performed a systematic review and meta-analysis of randomized controlled trials (RCTs) and their extension periods with the aim of estimating the incidence of OIs following treatment with b- and ts-DMARDs with different mechanisms of action (MOAs).

## 2 Methods

### 2.1 Data sources and search strategy

We searched the PubMed and EMBASE databases for RCTs published in English, with last access on 14 April 2022. For our literature search we used the following search term: “psoriatic arthritis” AND “randomized”. An additional manual search of reference lists for eligible studies complemented the initial search. We performed this meta-analysis based on the Preferred Reporting Items for Systematic Reviews and Meta-analyses (PRISMA) statement (Page et al., 2021).

### 2.2 Study selection

We selected RCTs of bDMARD or tsDMARD that compared the effect of a biologic or targeted synthetic agent with placebo and provided safety data. We decided to include in this analysis patients who received concomitant low-dose glucocorticoids, defined as <10 mg/day equivalent to prednisolone, and conventional synthetic DMARDs (csDMARDs) such as methotrexate, leflunomide and sulfasalazine. We excluded studies that randomized patients to two biologic agents with no placebo arm. Moreover, we marked data as unextractable and excluded studies that reported infectious causes of adverse events only with a high-level term and without any further categorization. Lastly, each outcome of interest had to be reported in ≥3 trials and the MOA of the tested treatment regimen had to be present in ≥3 trials.

### 2.3 Data extraction and quality assessment

Two reviewers (AV and GB) independently screened titles and abstracts to determine eligibility. The same reviewers independently retrieved and evaluated the full text of selected articles. They resolved disagreements through discussion and consensus; a third reviewer (FS) independently reviewed unresolved matters.

We independently extracted data regarding patient populations, interventions, outcomes of interest, and quality of data for individual studies. The extracted data included the main characteristics of each study (author and publication year, duration of RCT and extension period), proportion of bDMARD-naïve population, proportion of population with concomitant csDMARD use, proportion of women, number of patients in each treatment arm and placebo arm, and the number of patients that received at least one dose of an agent of interest. For our analysis, we also extracted the number of OIs and their causes during both the entire duration of follow-up and exclusively the placebo-controlled period and the number of herpes zoster and opportunistic *Candida* spp*.* infections during the entire duration of follow-up.

For methodological quality, we assessed the risk of bias of RCTs with the Revised Cochrane risk-of-bias tool by evaluating 1) the randomization process, 2) deviations from the intended interventions, 3) amount of missing outcome data, 4) measurement of the outcome, and 5) selection of the reported result (Sterne et al., 2019).

### 2.4 Definitions and outcomes

The primary outcome of our analysis was the incidence of all OIs stratified by MOA. We identified OIs based on the recommended definition of OIs for rheumatologic diseases by Winthrop et al. (2015). Secondary outcomes of our study were 1) incidence of herpes zoster infection by MOA, 2) incidence of opportunistic *Candida* spp*.* infections following anti-IL-17 treatment, 3) proportion of *M. tuberculosis* infections in patients with OIs receiving anti-TNFs and 4) the relative risk of OIs stratified by MOA during the placebo-controlled period.

The biologic agents evaluated include anti-TNFs (etanercept, infliximab, golimumab, certolizumab, and adalimumab), anti-IL-17 (ixekizumab, secukinumab, brodalumab, bimekizumab), anti-IL-12/23 (ustekinumab), anti-IL-23 (risankizumab, guselkumab), and T-cell co-stimulation modulators (abatacept, alefacept). The PDE4 inhibitor, apremilast and JAKi (tofacitinib, upadacitinib, filgotinib, deucravacitinib) were the targeted synthetic agents evaluated.

### 2.5 Statistical analysis

We used Stata v17 software (Stata Corporation, College Station, TX) for data analysis. We stratified by MOA of the agents tested and performed a random effects meta-analysis using the DerSimonian and Laird approach to estimate the cumulative incidence of OIs among patients with PsA during both placebo-controlled and extension periods (DerSimonian and Laird, 1986). In order to stabilize the variances, we used the Freeman Tukey double arcsine transformation (Nyaga et al., 2014). For this meta-analysis, we selected a random effects model due to differences in the proportion of bDMARD-naïve population, the proportion of concomitant csDMARD use, and duration of follow-up periods. Additionally, we conducted a meta-regression analysis to investigate the extent of the differences in study characteristics and their correlation with the heterogeneity between studies (Harbord and Higgins, 2008).

For our secondary analyses, we planned to stratify our data by the most common causes of OIs. We calculated a pooled random-effects estimate using the DerSimonian and Laird approach to estimate the cumulative incidence of our secondary outcomes, as well as the relative risk for OIs during the placebo-controlled period of RCTs (DerSimonian and Laird, 1986). We estimated heterogeneity using the I^2^ statistic and we used the Egger’s test to explore publication bias and small study effects (Higgins et al., 2003; Peters et al., 2006). For the interpretation of heterogeneity with the I2 statistic we used the approach detailed as follows: I2 values of 25%, 50%, and 75% represent low, moderate, and high heterogeneity, respectively (Higgins et al., 2003). Statistical significance was set at α = 0.05.

## 3 Results

### 3.1 Search results and study characteristics

**FIGURE 1:**
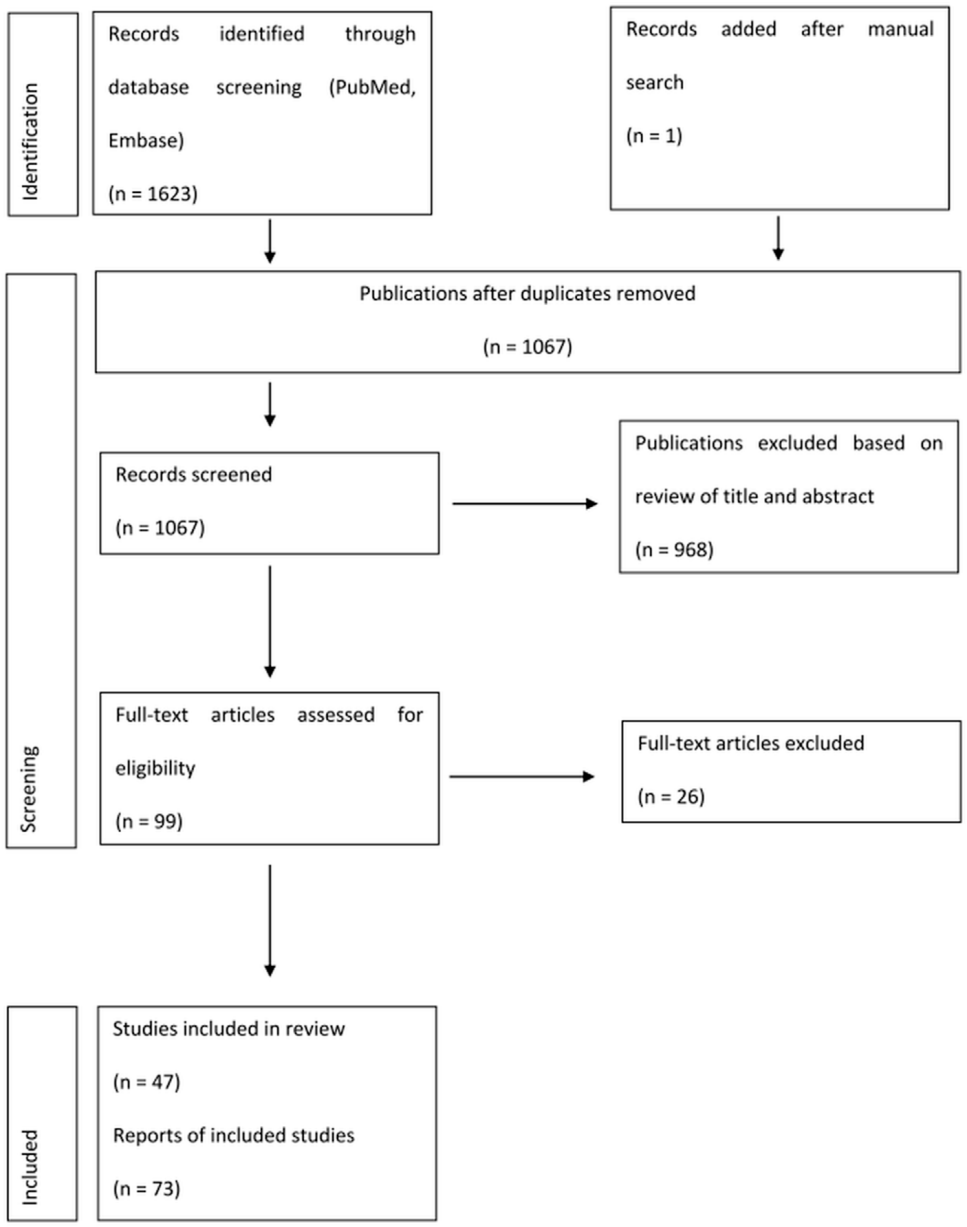
Flow diagram for selection of studies included in the systematic review and meta-analysis.

Following deduplication between literature search in PubMed and EMBASE, we retrieved 1,066 studies published between 2000 and 2022. One article was added after a manual search of reference lists. After title and abstract screening, we excluded a total of 968 publications and we retrieved 99 publications for full-text detailed evaluation. Subsequently, we excluded 26 publications, resulting in a total of 73 citations eligible for analysis. We retrieved 47 RCTs for this analysis and twenty-six studies reporting extension follow-up data (Figure 1, Supplementary Appendix Table S1).

Among the included studies, there were 17 studies evaluating anti-TNFs, 9 studies evaluating anti-IL-17, 6 studies each evaluating JAKi, anti-IL-23, and PDE4 inhibitors, and 3 studies each evaluating anti-IL-12/23 and cytotoxic T lymphocyte-associated antigen-4 Ig (CTLA4-Ig).

Regarding publications with extension periods, there were 11 studies for anti-TNFs, 5 studies for anti-IL-17, 4 studies for PDE4i, 3 studies for anti-IL-23, 2 studies for JAKi, 1 study for alefacept and 1 study for ustekinumab (Gladman et al., 2007; Kavanaugh et al., 2007; Antoni et al., 2008; Mease et al., 2009; Mease and Reich, 2009; Kavanaugh et al., 2012; Kavanaugh et al., 2013; Kavanaugh et al., 2014b; Kavanaugh et al., 2015a; Kavanaugh et al., 2015b; Mease et al., 2015b; Kavanaugh et al., 2017b; Genovese et al., 2018a; van der Heijde et al., 2018; Kavanaugh et al., 2019; Mease et al., 2020c; Husni et al., 2020; van der Heijde et al., 2020; McInnes et al., 2021b; Mease et al., 2021b; McInnes et al., 2021c; Mease et al., 2021c; Orbai et al., 2021; Ritchlin et al., 2021; McInnes et al., 2022; Wells et al., 2022). The baseline characteristics of the studies included are shown in the Supplementary Appendix Table S2.

In total, 11,790 patients were assigned to receive different doses of the tested agents and 6,425 patients were assigned to receive placebo during the placebo-controlled period (range: 12–48 weeks). After taking into consideration the extension periods, a total of 17,197 patients received at least one different dose of an agent for a total follow-up duration ranging from 12 to 268 weeks.

### 3.2 Opportunistic infections incidence based on MOA

#### 3.2.1 JAKi

**FIGURE 2:**
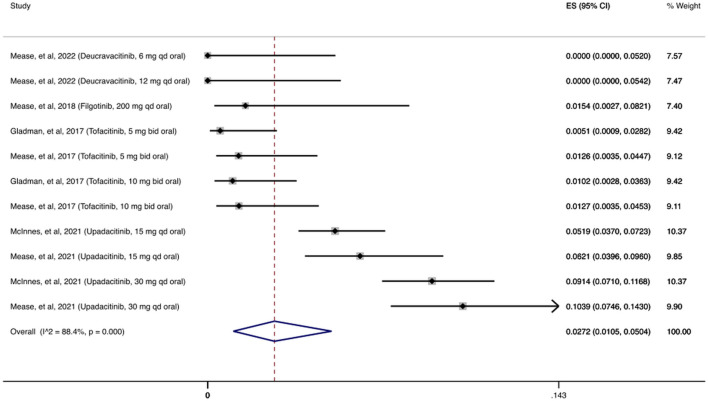
Opportunistic infections cumulative incidence for JAK inhibitors during RCTs and their extension periods. Individual and combined estimates of the cumulative incidence of opportunistic infections for patients treated with JAK inhibitors with 95% confidence intervals. ES: Effect Size (Cumulative incidence).

In Figure 2 we present the cumulative incidence of OIs for JAKi, which was 2.72% (95% CI: 1.05%–5.04%) among 2,740 patients receiving at least one dose of a JAKi agent. During the follow-up period (12–56 weeks), the most common OI reported following JAKi treatment was herpes zoster infection, with 130/146 (89%) patients with OIs developing herpes zoster infection.

#### 3.2.2 Anti-IL-17

**FIGURE 3:**
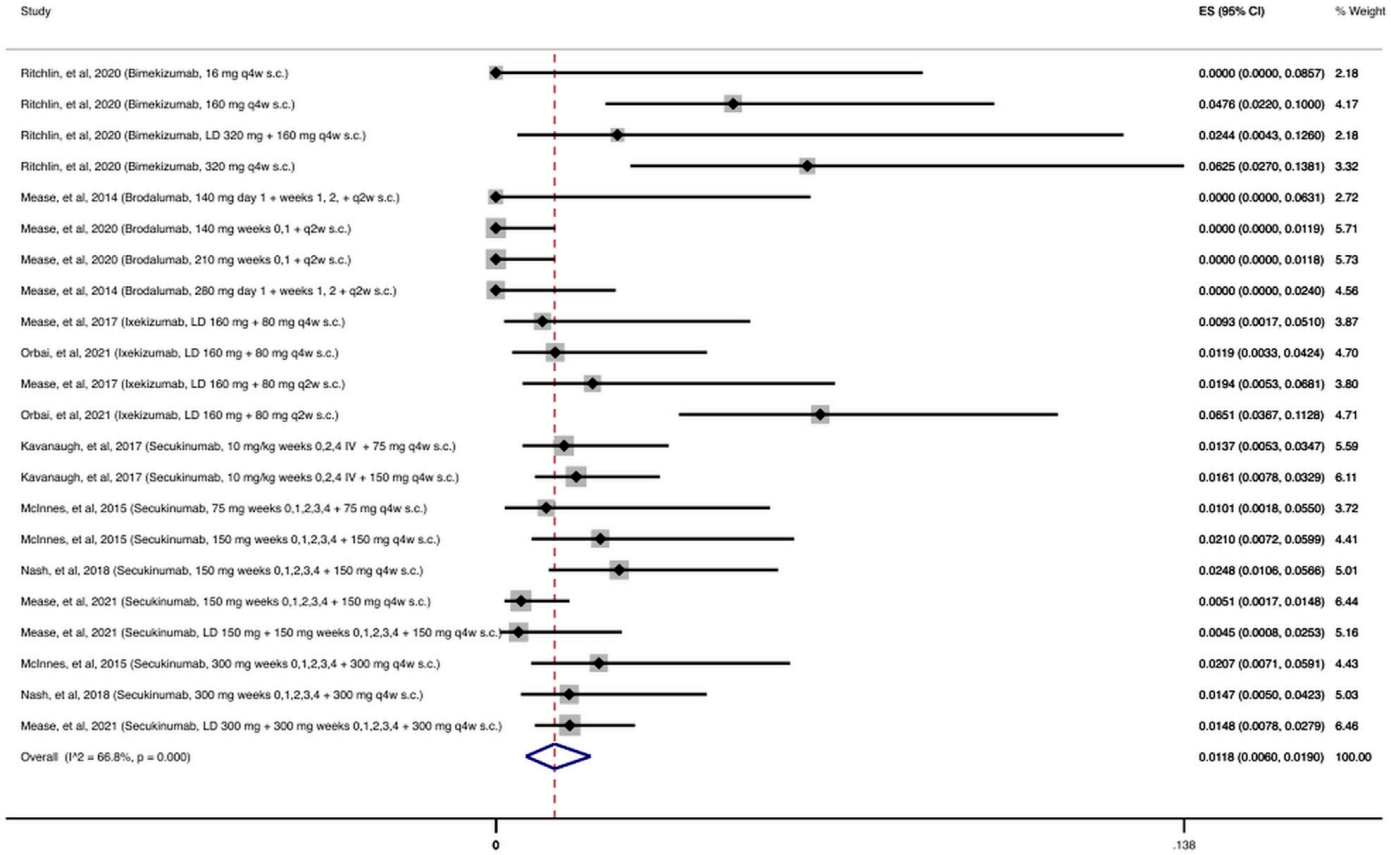
Opportunistic infections cumulative incidence for anti-IL-17 during RCTs and their extension periods. Individual and combined estimates of the cumulative incidence of opportunistic infections for patients treated with anti-IL-17 with 95% confidence intervals. ES: Effect Size (Cumulative incidence).

As shown in Figure 3, the cumulative incidence of OIs for anti-IL-17 was 1.18% (95% CI: 0.60%–1.9%) among 4,626 patients receiving at least one dose of an anti-IL-17 agent. The most common OI reported during the follow-up period (12–156 weeks) was due to *Candida* spp., with 58/67 (87%) patients with OIs developing an opportunistic *Candida* spp*.* infection.

#### 3.2.3 Anti-IL-23

**FIGURE 4:**
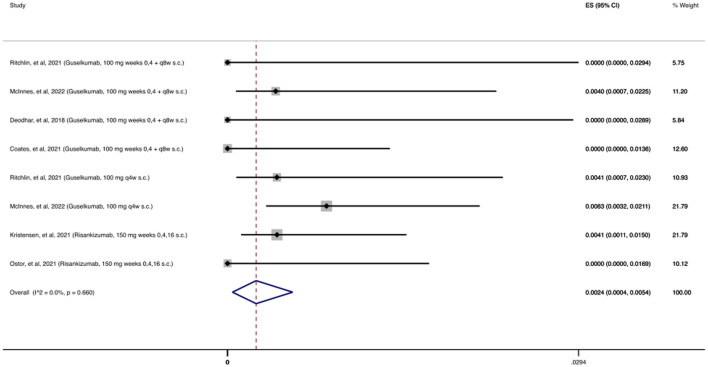
Opportunistic infections cumulative incidence for anti-IL-23 during RCTs and their extension periods. Individual and combined estimates of the cumulative incidence of opportunistic infections for patients treated with anti-IL-23 with 95% confidence intervals. ES: Effect Size (Cumulative incidence).

As shown in Figure 4, 2,215 patients were treated with at least one dose of an anti-IL-23 agent during the follow-up period (24–112 weeks). There were only 8 reported OIs, resulting in a cumulative incidence of 0.24% (95% CI: 0.04%–0.54%).

#### 3.2.4 Anti-TNFs

The cumulative incidence of OIs for anti-TNFs, as shown in the Supplementary Appendix Figure S1, was 0.01%, (95% CI: 0.00%–0.21%) among 3,425 patients receiving at least one dose of an anti-TNF agent during the follow-up period (12–268 weeks). More specifically, there were a total of 17 OIs reported, in which 5/17 OIs were due to *M. tuberculosis* infection. As shown in the Supplementary Appendix Figure S2, we found a 29.24% (95% CI: 29%–71.45%) pooled estimated proportion of *M. tuberculosis* infection in patients with OIs.

#### 3.2.5 Anti-IL-12/23, PDE4i, CTLA4**-**Ig

The cumulative incidence of OIs for these MOAs is presented in the Supplementary Appendix Figures S3–S5. Only one *Pneumocystis jirovecii* infection was reported among patients receiving abatacept and one herpes zoster infection among patients receiving apremilast. No OIs were noted among patients receiving anti-IL-12/23 agents.

We performed a meta-regression analysis for each group of agents based on their MOA and found no association between the cumulative incidence of OIs and the proportion of bDMARD-naïve population, the proportion of concomitant csDMARD use, and duration of follow-up (data not shown).

Besides herpes zoster*, Candida* spp. and *M. tuberculosis* infections, other causes of OIs were rarely reported as presented in the Supplementary Appendix Table S3. There were three MOAs under trial for the treatment of PsA that were evaluated by only one study each (Papp et al., 2007; Mease et al., 2016; Mease et al., 2018a; Genovese et al., 2018b). Of note, only two oral candidiasis infections were reported among 239 patients who received at least one dose of ABT-122, an agent targeting both TNF and IL-17A.

### 3.3 Secondary outcomes

#### 3.3.1 Incidence of herpes zoster infection

**FIGURE 5:**
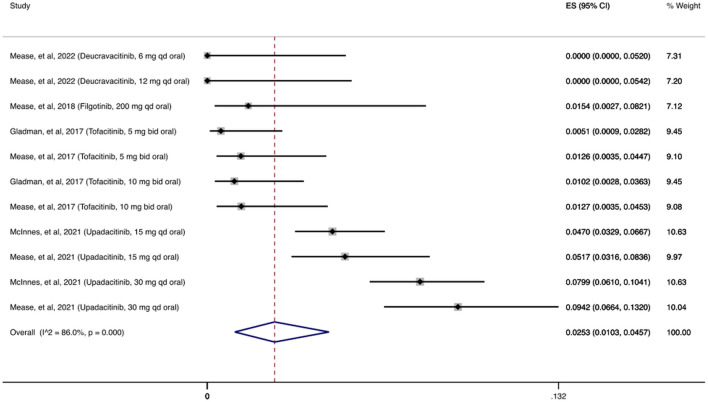
Herpes zoster infection cumulative incidence for JAK inhibitors during RCTs and their extension periods. Individual and combined estimates of the incidence of herpes zoster infection for patients treated with JAK inhibitors with 95% cumulative confidence intervals. ES: Effect Size (Cumulative incidence).

Based on the OIs consensus, all herpes zoster infections are adjudicated as OIs (Winthrop et al., 2015). As shown in Figure 5, the cumulative incidence of herpes zoster infection following treatment with JAKi was 2.53% (95% CI: 1.03%–4.57%). In the deucravacitinib study, an investigational agent selectively targeting tyrosine kinase 2 (TYK2 inhibitor), no cases of herpes zoster were reported (Mease et al., 2022). In contrast, upadacitinib studies had the highest number of herpes zoster infections (Mease et al., 2020a; McInnes et al., 2021a; McInnes et al., 2021b; Mease et al., 2021c; Burmester et al., 2022). Across the remaining studies evaluating the other MOAs, the incidence of herpes zoster infection was low with 14 cases among the combined 14,757 patients receiving at least one dose of the agents examined.

#### 3.3.2 Incidence of *Candida* spp infection

**FIGURE 6:**
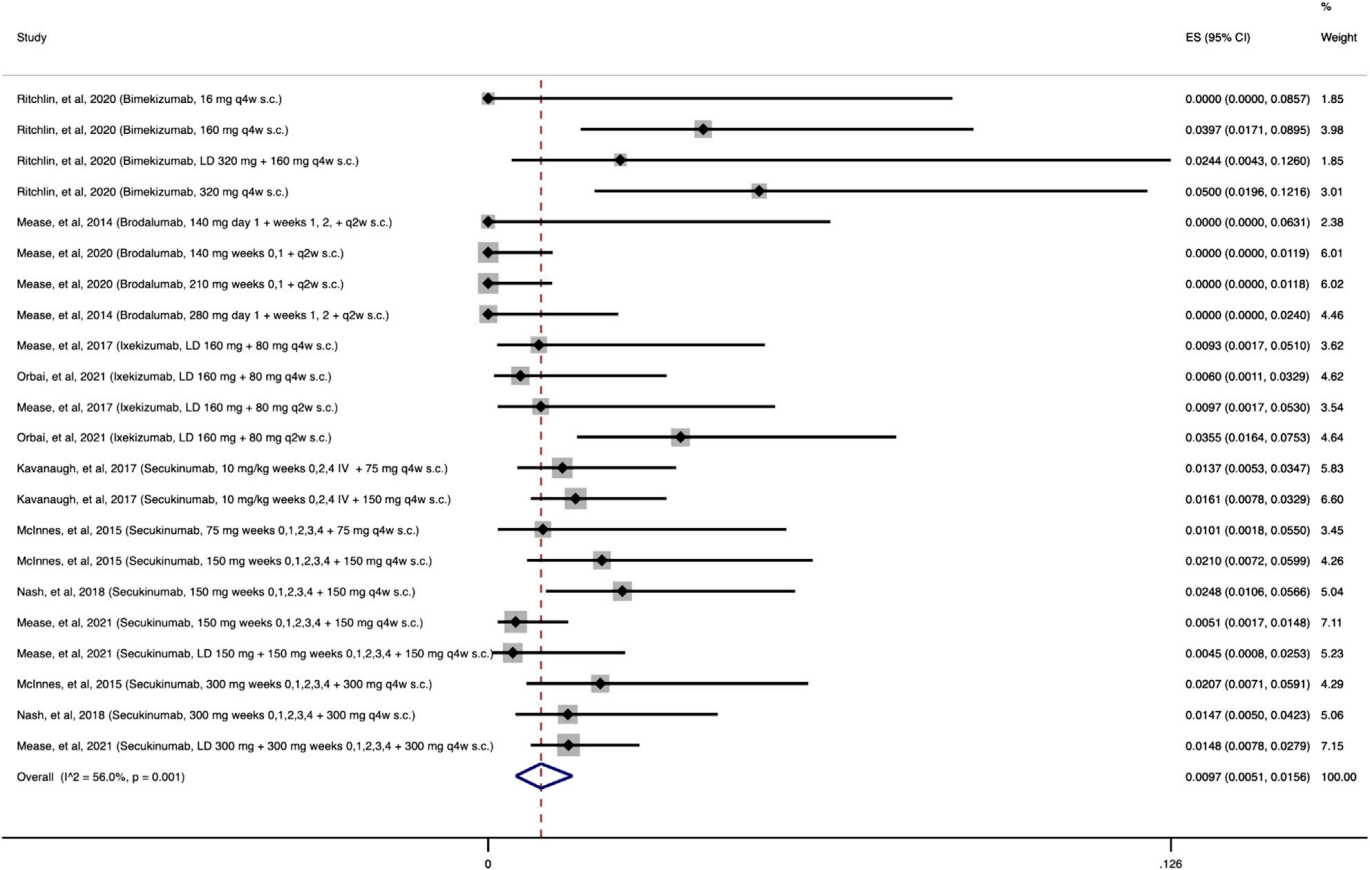
Opportunistic Candida spp. infections cumulative incidence for anti-IL-17 during RCTs and their extension periods. Individual and combined estimates of the cumulative incidence of opportunistic *Candida* spp. infections for patients treated with anti-IL-17 with 95% confidence intervals. ES: Effect Size (Cumulative incidence).

As shown in Figure 6, the cumulative incidence of opportunistic *Candida* spp. infections following treatment with anti-IL-17 was 0.97% (95% CI: 0.51%–1.56%). Most patients had mucocutaneous (oropharyngeal or esophageal) candidiasis. Across the remaining studies evaluating the other MOAs, 11 opportunistic *Candida* spp*.* infections were reported among the combined 12,468 patients receiving at least one dose of the agents of interest.

#### 3.3.3 Relative risk for OIs during the placebo-controlled period

In Table 1 **,** we show extracted data from studies specifically during the placebo-controlled period and our results stratified by MOA. Importantly, as shown in Supplementary Appendix Figures S6–S10 we detected no significant difference in the relative risk for OIs between patients treated with anti-TNFs, anti-IL-23, anti-IL-12/23, CTLA4-Ig, or a PDE4 inhibitor and those treated with a placebo. In contrast, patients treated with JAKi and anti-IL-17 agents had a 2.25 (95% CI: 1.16–4.35) and 2.27 (95% CI: 1.03–4.99) higher relative risk of OIs compared with patients treated with placebo, as shown in Supplementary Appendix Figures S11, S12, respectively. Moreover, most of the OIs reported among JAKi-treated patients (Cumulative incidence: 1.10%, 95% CI: 0.53%–1.83%) were due to herpes zoster infection (82.7%), while most of the OIs reported among anti-IL-17-treated patients (Cumulative incidence: 0.26%, 95% CI: 0.01%–0.70%) were due to *Candida* spp*.* (93.75%).

**TABLE 1:** Opportunistic infections cumulative incidence and relative risk (RR) for bDMARDs, tsDMARDs during placebo-controlled period.

Mechanism of action	No of studies	No of patients	No of placebo	Range of follow-up (weeks)	RR	95% CI	Cumulative incidence %	95% CI
TNF inhibitors	17	2621	1984	12–48	0.87	0.37–2.01	0.00	0.00–0.00
IL-17 inhibitors	9	2578	1312	12–24	2.27	1.03–4.99	0.26	0.01–0.70
JAK inhibitors	6	1957	1003	12–24	2.25	1.16–4.35	1.10	0.53–1.83
IL-23 inhibitors	6	1744	1217	24	0.88	0.26–3.02	0.02	0.00–0.25
PDE4 inhibitors	6	1595	848	12–24	1.00	0.31–3.24	0.00	0.00–0.04
IL-12/23 inhibitors	3	693	380	12–24	0.99	0.17–5.67	0.00	0.00–0.27
CTLA4-Ig	3	464	315	12–24	1.18	0.22–6.23	0.02	0.00–0.66

### 3.4 Heterogeneity and quality of individual studies

Studies with PDE4 inhibitors, anti-IL-12/23, anti-IL-23 and CTLA4-Ig had low heterogeneity (I^2^ = 0%, *p* > 0.05). Anti-TNFs showed moderate heterogeneity (I^2^ = 31.69%, *p* < 0.05), while anti-IL-17 and JAKi had high heterogeneity (I^2^ = 66.8%, *p* = 0.00 and I^2^ = 88.4%, *p* = 0.00, respectively)

We present quality assessment data in the Supplementary Appendix Figure S13. We considered all of the studies to have low risk of bias across all domains evaluated. Also, Egger’s test for publication bias showed no evidence of small-study effects (bias = 0.014, *p* = 0.67).

## 4 Discussion

New biologic and targeted synthetic disease-modifying agents are becoming increasingly available for the treatment of PsA. Our meta-analysis of almost 17,000 patients treated with different b- or ts-DMARDs across RCTs and their extension periods estimated the cumulative incidence of OIs stratified by MOA. It should be noted that we excluded studies without a placebo arm. Interestingly, we found that the cumulative incidence of OIs was low and that the most common OI differed based on the drug MOA. The randomized nature of the included studies and the lack of statistical heterogeneity in many analyses strengthen our findings, which offer insight about the safety and incidence of OIs in daily clinical practice and highlight the need for careful monitoring of patients treated with these agents for OIs.

In our analysis, the cumulative incidence of OIs was less than 3% across all MOAs examined. Our findings are in line with published OI incidences in real-world studies, which identify the same predicted causes for each MOA, various rates according to the MOA, and minimal risk for severe adverse outcomes (Siegel and Winthrop, 2019; Li et al., 2020). The low incidence may be attributed to the selection of more homogenous populations across RCTs and thorough screening for latent TB and other infections prior to treatment initiation (Ogdie and Coates, 2017). Additionally, short-term follow-up periods and acquired experience for OIs monitoring during therapy may have also influenced the rate of reported OIs. Follow-up periods of placebo-controlled RCTs usually lasted up to 24 weeks. However, most OIs occurred during the extension period and long-term placebo is questionable ethically (Ogdie and Coates, 2017). Therefore, more RCTs with longer follow-up periods and head-to-head comparisons of b- and ts-DMARDs are needed.

Herpes zoster infection was the most common OI among patients treated with JAKi. The cumulative incidence of herpes zoster infection was almost 2.5% in JAKi-treated patients. Age, comorbidities, and the effect of JAKi on T cell function and inhibition of IFN-γ and IL-15 are potential risk factors for varicella zoster virus reactivation (McLornan et al., 2015; Choy, 2019; Sunzini et al., 2020).

Among the 130 herpes zoster infections reported, we observed rare occurrence of disseminated herpes zoster or permanent drug discontinuation due to herpes zoster infection. There were higher rates of herpes zoster infection following treatment with upadacitinib among patients receiving at least one dose of 30 mg qd (8.4%), with this dose not currently approved for treatment of PsA, compared with patients receiving at least one dose of 15 mg qd (4.8%), which is approved for PsA treatment (McInnes et al., 2021b; Mease et al., 2021c). Interestingly, absence of herpes zoster infection in the TYK2 inhibitor study may explain the substantial heterogeneity in the reported outcomes of JAK family inhibitors. TYK2 inhibitors need further studies since the activity of TYK2 plays a major role in the occurrence of psoriatic skin lesions and pathological synovial response (Gao et al., 2016; Mease et al., 2022). Compared with our incidence, the incidence of herpes zoster infection is higher among rheumatoid arthritis (RA) patients treated with tofacitinib and upadacitinib (Cohen et al., 2020; Álvaro-Gracia et al., 2021). The mean incidence rate for herpes zoster infection among patients with RA receiving tofacitinib was 4.1 (95% CI: 3.3–5.2) at a dose of 10 mg qd and 3.3 (95% CI: 2.6–4.3) at a dose of 5 mg qd (Álvaro-Gracia et al., 2021). Higher rates of herpes zoster infection could be explained by the different comorbidities of RA and use of glucocorticoids to treat this disease, while glucocorticoids are sparingly used for PsA (Winthrop et al., 2017).

We found mucocutaneous candidiasis as the most common OI among patients treated with anti-IL-17. The severity of *Candida* spp*.* infections was either mild or moderate in patients receiving anti-IL-17 treatment. The incidence of opportunistic *Candida* spp. infections was 0.97%, which is in concordance with previous research (Saunte et al., 2017). Anti-IL-17 therapies, by either blocking the IL-17 receptor or IL-17A and/or IL-17F homodimers or heterodimers, increase the incidence of *Candida* spp*.* infections (Ghoreschi et al., 2021). Patients with inherited deficiencies in the IL-17 pathway (e.g., IL-17RA, IL-17RC, or IL-17F gene mutations) are at higher risk for developing chronic mucocutaneous candidiasis, but do not often develop systemic, disseminated or invasive candidiasis (Puel et al., 2011). Similarly, patients from our included studies developed mucocutaneous candidiasis, which was mostly oropharyngeal. In the majority of cases, discontinuation of anti-IL-17 treatment was not necessary because of monitoring, proper treatment, and adequate treatment response.

Psoriatic arthritis can be effectively treated with anti-TNF agents and disease development, disease severity, and response to anti-TNFs, particularly etanercept and adalimumab, appear to be influenced by several single-nucleotide polymorphisms (Murdaca et al., 2014; Murdaca et al., 2017). Therefore, pharmacogenetic testing of polymorphisms of TNF and TNF receptor, Fc receptors, and IL-17, and HLA gene variants could all be potential predictors of treatment response (Murdaca et al., 2014; Murdaca et al., 2017). The incidence of OIs in anti-TNF treated patients was 0.01%. This comes in contrast to data from a study that assessed the effect of anti-TNF agents on OIs among patients with RA (Kourbeti et al., 2014). Anti-TNF treated patients with RA were more likely to develop an OI (Kourbeti et al., 2014). Nevertheless, in our study, 5 cases of *M. tuberculosis* infection among well-screened and monitored patients with PsA were reported, emphasizing the need for increased surveillance during anti-TNF treatment.

Regarding study limitations, we could not pinpoint a specific time frame (e.g., week of infection) as when OIs developed. Also, not all published trials followed a universal definition of OIs, such as the consensus suggested by Winthrop et al. (2015). Moreover, we could not assess the risk of OIs in comparison to available treatment options, so further head-to-head studies are needed to determine the risk. Lastly, lengthier study periods are needed to assess the risk of uncommon OIs, especially OIs with prolonged latent periods. Thus, continued evaluation of the incidence of OIs following treatment with b- or ts-DMARDs is encouraged through post-marketing studies and clinical trials with longer follow-up periods.

## 5 Conclusion

This is the largest meta-analysis to date that evaluated the incidence of OIs in patients with PsA. Data from our meta-analysis indicate that both biologic and targeted synthetic DMARDs are safe agents concerning OIs for the treatment of PsA, since the incidence of OIs was found to be low across various different agents. However, due to the increasing use of these biologic agents in clinical settings, it is important to continue thorough monitoring of patients with PsA who are treated with biologic and targeted synthetic DMARDs, particularly for herpes zoster infection in patients treated with JAKi, mucocutaneous candidiasis in patients treated with anti-IL-17, and *M. tuberculosis* infection in patients treated with anti-TNFs.

## Data Availability

The original contributions presented in the study are included in the article/Supplementary Material, further inquiries can be directed to the corresponding author.

## Abbreviations

CI, confidence intervals; cs, conventional synthetic; CTLA4-Ig, cytotoxic T lymphocyte-associated antigen-4 Ig; DMARD, disease-modifying anti-rheumatic drug; IL, interleukin; JAK, Janus kinase; MOA, mechanism of action; OI, Opportunistic infection; PDE, phosphodiesterase; PsA, psoriatic arthritis; RCT, randomized controlled trial; TNF, tumor necrosis factor; ts, targeted synthetic.
