# Supplementary material for: The incidence of opportunistic infections in patients with psoriatic arthritis treated with biologic and targeted synthetic agents: A systematic review and meta-analysis
Source: Front Pharmacol. 2022 Oct 5;13:992713. doi: 10.3389/fphar.2022.992713 (PMC9579334; doi:10.3389/fphar.2022.992713)
Supplement: Supplementary file 1 [file DataSheet1.PDF]

Supplementary Appendix

Supplement to: **The Incidence of Opportunistic Infections in Patients with Psoriatic Arthritis Treated with Biologic and Targeted Synthetic Agents: A Systematic Review and Meta-analysis**

Contents

Section 1: Supplemental Tables and Figures
Table S1: PRISMA table checklist
Table S2: Study characteristics
Table S3: Rare causes of reported opportunistic infections
Figure S1: Opportunistic infections cumulative incidence for anti-TNFs during RCTs and their extension periods
Figure S2: Pooled estimated proportion of Mycobacterium tuberculosis infections in patients with opportunistic infections receiving anti-TNFs
Figure S3: Opportunistic infections cumulative incidence for T-cell co-stimulation modulators during RCTs and their extension periods
Figure S4: Opportunistic infections cumulative incidence for anti-IL-12/23 during RCTs and their extension periods
Figure S5: Opportunistic infections cumulative incidence for PDE4 inhibitors during RCTs and their extension periods
Figure S6: Relative risk of anti-TNFs for opportunistic infections compared to placebo during placebo controlled period
Figure S7: Relative risk of anti-IL-23 for opportunistic infections compared to placebo during placebo controlled period
Figure S8: Relative risk of anti-IL-12/23 for opportunistic infections compared to placebo during placebo controlled period
Figure S9: Relative risk of T-cell co-stimulation modulators for opportunistic infections compared to placebo during placebo controlled period
Figure S10: Relative risk of PDE4 inhibitors for opportunistic infections compared to placebo during placebo controlled period
Figure S11: Relative risk of JAK inhibitors for opportunistic infections compared to placebo during placebo controlled period
Figure S12: Relative risk of anti-IL-17 inhibitors for opportunistic infections compared to placebo during placebo controlled period
Figure S13: Individual study risk of bias in each domain and overall

Section 1: Supplemental Tables and Figures

Table S1 PRISMA table checklist

Topic	No.	Item	Location where item is reported
**TITLE**			
**Title**	1	Identify the report as a systematic review.	Lines 1-3
**ABSTRACT**			
**Abstract**	2	See the PRISMA 2020 for Abstracts checklist.	
**INTRODUCTION**			
**Rationale**	3	Describe the rationale for the review in the context of existing knowledge.	Lines 71-75
**Objectives**	4	Provide an explicit statement of the objective(s) or question(s) the review addresses.	Lines 114-127
**METHODS**			
**Eligibility criteria**	5	Specify the inclusion and exclusion criteria for the review and how studies were grouped for the syntheses.	Lines 84-94

Topic	No.	Item	Location where item is reported
**Information sources**	6	Specify all databases, registers, websites, organisations, reference lists and other sources searched or consulted to identify studies. Specify the date when each source was last searched or consulted.	Lines 77-83
**Search strategy**	7	Present the full search strategies for all databases, registers and websites, including any filters and limits used.	Lines 77-83
**Selection process**	8	Specify the methods used to decide whether a study met the inclusion criteria of the review, including how many reviewers screened each record and each report retrieved, whether they worked independently, and if applicable, details of automation tools used in the process.	Lines 84-94
**Data collection process**	9	Specify the methods used to collect data from reports, including how many reviewers collected data from each report, whether they worked independently, any processes for obtaining or confirming data from study investigators, and if applicable, details of automation tools used in the process.	Lines 95-113
**Data items**	10a	List and define all outcomes for which data were sought. Specify whether all results that were compatible with each outcome domain in each study were sought (e.g. for all measures, time points, analyses), and if not, the methods used to decide which results to collect.	Lines 114-127

Topic	No.	Item	Location where item is reported
	10b	List and define all other variables for which data were sought (e.g. participant and intervention characteristics, funding sources). Describe any assumptions made about any missing or unclear information.	Lines 95-113
**Study risk of bias assessment**	11	Specify the methods used to assess risk of bias in the included studies, including details of the tool(s) used, how many reviewers assessed each study and whether they worked independently, and if applicable, details of automation tools used in the process.	Lines 110-113
**Effect measures**	12	Specify for each outcome the effect measure(s) (e.g. risk ratio, mean difference) used in the synthesis or presentation of results.	Lines 128-148
**Synthesis methods**	13a	Describe the processes used to decide which studies were eligible for each synthesis (e.g. tabulating the study intervention characteristics and comparing against the planned groups for each synthesis (item 5)).	Lines 95-113
	13b	Describe any methods required to prepare the data for presentation or synthesis, such as handling of missing summary statistics, or data conversions.	N/A
	13c	Describe any methods used to tabulate or visually display results of individual studies and syntheses.	Lines 129-130

Topic	No.	Item	Location where item is reported
	13d	Describe any methods used to synthesize results and provide a rationale for the choice(s). If meta-analysis was performed, describe the model(s), method(s) to identify the presence and extent of statistical heterogeneity, and software package(s) used.	Lines 128-148
	13e	Describe any methods used to explore possible causes of heterogeneity among study results (e.g. subgroup analysis, meta-regression).	Lines 128-148
	13f	Describe any sensitivity analyses conducted to assess robustness of the synthesized results.	N/A
**Reporting bias assessment**	14	Describe any methods used to assess risk of bias due to missing results in a synthesis (arising from reporting biases).	N/A
**Certainty assessment**	15	Describe any methods used to assess certainty (or confidence) in the body of evidence for an outcome.	Lines 128-148
**RESULTS**			
**Study selection**	16a	Describe the results of the search and selection process, from the number of records identified in the search to the number of studies included in the review, ideally using a flow diagram.	Figure 1
	16b	Cite studies that might appear to meet the inclusion criteria, but which were excluded, and explain why they were excluded.	N/A

Topic	No.	Item	Location where item is reported
**Study characteristics**	17	Cite each included study and present its characteristics.	Lines 158-166
**Risk of bias in studies**	18	Present assessments of risk of bias for each included study.	Lines 243-246
**Results of individual studies**	19	For all outcomes, present, for each study: (a) summary statistics for each group (where appropriate) and (b) an effect estimate and its precision (e.g. confidence/credible interval), ideally using structured tables or plots.	Figures 2-6 and S1-S12
**Results of syntheses**	20a	For each synthesis, briefly summarise the characteristics and risk of bias among contributing studies.	Lines 243-246
	20b	Present results of all statistical syntheses conducted. If meta-analysis was done, present for each the summary estimate and its precision (e.g. confidence/credible interval) and measures of statistical heterogeneity. If comparing groups, describe the direction of the effect.	Figures 2-6 and S1-S12
	20c	Present results of all investigations of possible causes of heterogeneity among study results.	Lines 239-242
	20d	Present results of all sensitivity analyses conducted to assess the robustness of the synthesized results.	N/A
**Reporting biases**	21	Present assessments of risk of bias due to missing results (arising from reporting biases) for each synthesis assessed.	N/A

Topic	No.	Item	Location where item is reported
**Certainty of evidence**	22	Present assessments of certainty (or confidence) in the body of evidence for each outcome assessed.	Figures 2-6 and S1-S12
**DISCUSSION**			
**Discussion**	23a	Provide a general interpretation of the results in the context of other evidence.	Lines 248-269
	23b	Discuss any limitations of the evidence included in the review.	Lines 318-326
	23c	Discuss any limitations of the review processes used.	Lines 318-326
	23d	Discuss implications of the results for practice, policy, and future research.	Lines 318-326
**OTHER INFORMATION**			
**Registration and protocol**	24a	Provide registration information for the review, including register name and registration number, or state that the review was not registered.	N/A
	24b	Indicate where the review protocol can be accessed, or state that a protocol was not prepared.	N/A
	24c	Describe and explain any amendments to information provided at registration or in the protocol.	N/A
**Support**	25	Describe sources of financial or non-financial support for the review, and the role of the funders or sponsors in the review.	Line 769

Topic	No.	Item	Location where item is reported
**Competing interests**	26	Declare any competing interests of review authors.	Lines 758-760
**Availability of data, code and other materials**	27	Report which of the following are publicly available and where they can be found: template data collection forms; data extracted from included studies; data used for all analyses; analytic code; any other materials used in the review.	N/A

Table S2 Study characteristics

Study	Study last extension	Name of study	Duration total (weeks)	Population	bDMARD-naïve %	Concomitant csDMARD %	Age (mean)	Female (%)	Number of patients	Drug and doses
Mease, et al, 2011			24	Mixed	64	67	50.3± 9.9	51	45	Abatacept 3 mg/kg days 1,15,29 + 10 mg/kg q4w IV
Mease, et al, 2011			24	Mixed	67	63	50.8±10.5	35	40	Abatacept 10 mg/kg days 1,15,29 + 10 mg/kg q4w IV
Mease, et al, 2011			24	Mixed	49	60	51.5±9.8	54	43	Abatacept 30 mg/kg days 1,15 + 10 mg/kg day 29 + 10 mg/kg q4w IV
Mease, et al, 2017		ASTRAEA	52	Mixed	39.4	73.3	51.0±10.7	56.8	213	Abatacept 125 mg qw s.c.
Mease, et al, 2018	Genovese, et al, 2018		12	Mixed		100	50.5±12.0	45.8	72	Adalimumab 40 mg q2w s.c.
Genovese, et al, 2007			24	bDMARD-naïve	100	64.7	50.4±11.0	43.1	51	Adalimumab 40 mg q2w s.c.
Mease, et al, 2005	Mease, et al, 2009	ADEPT	144	bDMARD-naïve	100	51	48.6±12.5	43.7	151	Adalimumab 40 mg q2w s.c.
Mease, et al, 2006	Mease, et al, 2009		48	Mixed		100	45.6	50	123	Alefacept 15 mg qw IM 12 weeks / 12 weeks of MTX
Nash, et al, 2018		ACTIVE	52	bDMARD-naïve	100	0	50.7±12.2	52.7	110	Apremilast 30 mg bid oral
Schett, et al, 2012			28	Mixed		43.5	50.9	37.7	69	Apremilast 20 mg bid oral
Schett, et al, 2012			28	Mixed		44.8	49.9	52.2	67	Apremilast 40 mg qd oral
	Kavanaugh, et al, 2019	PALACE 1-3	260	Mixed	77	65.7	49.8±11.7	53.7	501	Apremilast 20 mg bid oral
	Kavanaugh, et al, 2019	PALACE 1-3	260	Mixed	78.5	64.2	50.6±11.4	55.3	497	Apremilast 30 mg bid oral
Wells, et al, 2018	Wells, et al, 2022	PALACE 4	260	bDMARD-naïve	100	0	49.2±12.0	54.3	175	Apremilast 20 mg bid oral
Wells,	Wells,		260	bDMARD-naïve	100	0	48.4±12.5	54.5	176	Apremilast

Study	Study last extension	Name of study	Duration total (weeks)	Population	bDMARD-naïve %	Concomitant csDMARD %	Age (mean)	Female (%)	Number of patients	Drug and doses
et al, 2018	et al, 2022	PALACE 4								30 mg bid oral
Ritchlin, et al, 2020		BE ACTIVE	12	Mixed	83	59	50.0±13.6	41	41	Bimekizumab 16 mg q4w s.c.
Ritchlin, et al, 2020		BE ACTIVE	48	Mixed	80	73	48.0±11.7	51	41	Bimekizumab 160 mg q4w s.c.
Ritchlin, et al, 2020		BE ACTIVE	12	Mixed	83	68	49.1±13.0	66	41	Bimekizumab LD 320 mg + 160 mg q4w s.c.
Ritchlin, et al, 2020		BE ACTIVE	48	Mixed	80	68	50.4±12.1	44	41	Bimekizumab 320 mg q4w s.c.
Mease, et al, 2014			12	Mixed	47	54	53.0±10.0	65	57	Brodalumab 140 mg day 1 + weeks 1, 2, + q2w s.c.
Mease, et al, 2014			52	Mixed	45	54	51.0±12.0	71	56	Brodalumab 280 mg day 1 + weeks 1, 2 + q2w s.c.
Mease, et al, 2014	van der Heijde, et al, 2018	RAPID-PsA	216	Mixed	77.5	71.7	48.2±12.3	53.6	138	Certolizumab LD 400 mg weeks 0,2,4 + 200mg q2w s.c.
Mease, et al, 2014	van der Heijde, et al, 2018	RAPID-PsA	216	Mixed	83	74.1	47.1±10.8	54.1	135	Certolizumab LD 400 mg weeks 0,2,4 + 400mg q4w s.c.
Mease, et al, 2022			16	Mixed	82.9	64.3	50.5±13.7	42.9	70	Deucravacitinib 6 mg qd oral
Mease, et al, 2022			16	Mixed	86.6	64.2	50.5±13.8	50.7	67	Deucravacitinib 12 mg qd oral
Mease, et al, 2000			12	bDMARD-naïve	100	47	46.0 (median)	47	30	Etanercept 25 mg biw s.c.
Mease, et al, 2004			24	bDMARD-naïve	100	42	47.6	43	101	Etanercept 25 mg biw s.c.
Mease, et al, 2019		SEAM-PsA	48	bDMARD-naïve	100	100	48.1±12.7	49.1	283	Etanercept 50 mg qw s.c.
Mease, et al, 2019		SEAM-PsA	48	bDMARD-naïve	100	0	48.5±13.5	46.8	284	Etanercept 50 mg qw s.c.

Study	Study last extension	Name of study	Duration total (weeks)	Population	bDMARD-naïve %	Concomitant csDMARD %	Age (mean)	Female (%)	Number of patients	Drug and doses
Mease, et al, 2018		EQUATOR	16	Mixed	83	72	49.0±12.2	55	65	Filgotinib 200 mg qd oral
Vieira-Sousa, et al, 2020		GO-DACT	24	bDMARD-naïve	100	100	46.2 (median)	19	21	Golimumab 50 mg q4w s.c.
Kavanaugh, et al, 2009	Kavanaugh, et al, 2014	GO-REVEAL	268	bDMARD-naïve	100	49	45.7±10.7	39	146	Golimumab 50 mg q4w s.c.
Kavanaugh, et al, 2009	Kavanaugh, et al, 2014	GO-REVEAL	268	bDMARD-naïve	100	47	48.2±10.9	41	146	Golimumab 100 mg q4w s.c.
Kavanaugh, et al, 2017	Husni, et al, 2020	GO-VIBRANT	60	bDMARD-naïve	100	67.6	45.7±11.3	46.9	240	Golimumab 2 mg/kg weeks 0,4 + q8w IV
J van Mens, et al, 2019			22	bDMARD-naïve	100	100	47.5±11.8	30.8	26	Golimumab 50 mg q4w s.c.
Coates, et al, 2021	Coates, et al, 2021	COSMOS	56	bDMARD-IR	0	56	49.0±12.0	54	189	Guselkumab 100 mg weeks 0,4 + q8w s.c.
Deodhar, et al, 2020	Ritchlin, et al, 2021	DISCOVER-1	60	Mixed	70	64	47.4±11.6	48	128	Guselkumab 100 mg q4w s.c.
Deodhar, et al, 2020	Ritchlin, et al, 2021	DISCOVER-1	60	Mixed	68	65	48.9±11.5	46	127	Guselkumab 100 mg weeks 0,4 + q8w s.c.
Mease, et al, 2020	McInnes, et al, 2022	DISCOVER-2	112	100	69	45.9±11.5	42	245	Guselkumab 100 mg q4w s.c.	
Mease, et al, 2020	McInnes, et al, 2022	DISCOVER-2	112	bDMARD-naïve	100	69	44.9±11.9	48	248	Guselkumab 100 mg weeks 0,4 + q8w s.c.
Deodhar, et al, 2018			56	Mixed	91	47	47.4±12.8	48	100	Guselkumab 100 mg weeks 0,4 + q8w s.c.
Antoni, et al, 2005	Antoni, et al, 2008	IMPACT	98	bDMARD-naïve	100	63	45.7±11.1	42.3	52	Infliximab 5 mg/kg weeks 0,2,6 + q8w IV
Antoni, et al, 2005	Kavanaugh, et al, 2007	IMPACT 2	54	bDMARD-naïve	100	47	47.1±12.8	29	100	Infliximab 5 mg/kg weeks 0,2,6 + q8w IV
Mease, et al, 2017		SPIRIT-P1	24	bDMARD-naïve	100	61.2	49.8±12.6	53.4	103	Ixekizumab LD 160 mg + 80 mg q2w s.c.
Mease,			24	bDMARD-naïve	100	63.6	49.1±10.1	57.9	107	Ixekizumab

Study	Study last extension	Name of study	Duration total (weeks)	Population	bDMARD-naïve %	Concomitant csDMARD %	Age (mean)	Female (%)	Number of patients	Drug and doses
et al, 2017		SPIRIT-P1								LD 160 mg + 80 mg q4w s.c.
Mease, et al, 2017		SPIRIT-P1	24	bDMARD-naïve	100	66.3	48.6±12.4	49.5	101	Adalimumab 40 mg q2w s.c.
Nash, et al, 2017	Orbai, et al, 2021	SPIRIT-P2	156	bDMARD-IR	0	59	51.7±11.9	59	123	Ixekizumab LD 160 mg + 80 mg q2w s.c.
Nash, et al, 2017	Orbai, et al, 2021	SPIRIT-P2	156	bDMARD-IR	0	49	52.6±13.6	48	122	Ixekizumab LD 160 mg + 80 mg q4w s.c.
Kristensen, et al, 2021		KEEPsAKE 1	24	bDMARD-naïve	100	75.8	52 (median)	47.8	483	Risankizumab 150 mg weeks 0,4,16 s.c.
Ostor, et al, 2021		KEEPsAKE 2	24	bDMARD-IR	0	62.9	53 (median)	55.4	224	Risankizumab 150 mg weeks 0,4,16 s.c.
Mease, et al, 2015	Kavanaugh, et al, 2017	FUTURE 1	104	Mixed	70.3	60.4	48.8±12.2	58.4	202	Secukinumab 10 mg/kg weeks 0,2,4 IV + 75 mg q4w s.c.
Mease, et al, 2015	Kavanaugh, et al, 2017	FUTURE 1	104	Mixed	70.8	59.9	49.6±11.8	52.5	202	Secukinumab 10 mg/kg weeks 0,2,4 IV + 150 mg q4w s.c.
McInnes, et al, 2015		FUTURE 2	52	Mixed	66	47	48.6±11.4	53	99	Secukinumab 75 mg weeks 0,1,2,3,4 + 75 mg q4w s.c.
McInnes, et al, 2015		FUTURE 2	52	Mixed	63	44	46.5±11.7	45	100	Secukinumab 150 mg weeks 0,1,2,3,4 + 150 mg q4w s.c.
McInnes, et al, 2015		FUTURE 2	52	Mixed	67	44	46.9±12.6	49	100	Secukinumab 300 mg weeks 0,1,2,3,4 + 300 mg q4w s.c.
Nash, et al, 2018		FUTURE 3	52	Mixed	68.1	42.8	50.1±11.7	55.8	138	Secukinumab 150 mg weeks 0,1,2,3,4 + 150 mg q4w s.c.
Nash, et al, 2018		FUTURE 3	52	Mixed	68.3	50.4	49.3±12.9	51.8	139	Secukinumab 300 mg weeks 0,1,2,3,4 + 300 mg q4w s.c.
Mease, et al, 2018	Mease, et al, 2021	FUTURE 5	104	Mixed	71.2	54.1	48.8±11.8	45.9	222	Secukinumab 150 mg weeks 0,1,2,3,4 + 150 mg q4w s.c.

Study	Study last extension	Name of study	Duration total (weeks)	Population	bDMARD-naïve %	Concomitant csDMARD %	Age (mean)	Female (%)	Number of patients	Drug and doses
Mease, et al, 2018	Mease, et al, 2021	FUTURE 5	24	Mixed	70.5	49.1	48.4±12.9	49.5	220	Secukinumab LD 150 mg + 150 mg weeks 0,1,2,3,4 + 150 mg q4w s.c.
Mease, et al, 2018	Mease, et al, 2021	FUTURE 5	104	Mixed	69.4	50.5	48.9±12.8	51.4	222	Secukinumab LD 300 mg + 300 mg weeks 0,1,2,3,4 + 300 mg q4w s.c.
Gladman, et al, 2017		OPAL Beyond	24	bDMARD-IR	0	100	49.5±12.3	49	131	Tofacitinib 5 mg bid oral
Gladman, et al, 2017		OPAL Beyond	24	bDMARD-IR	0	100	51.3±10.9	56	132	Tofacitinib 10 mg bid oral
Mease, et al, 2017		OPAL Broaden	52	bDMARD-naïve	100	100	49.4±12.6	53	107	Tofacitinib 5 mg bid oral
Mease, et al, 2017		OPAL Broaden	52	bDMARD-naïve	100	100	46.9±12.4	60	104	Tofacitinib 10 mg bid oral
Mease, et al, 2017		OPAL Broaden	52	bDMARD-naïve	100	100	47.4±11.3	47	106	Adalimumab 40 mg q2w s.c.
McInnes, et al, 2021	McInnes, et al, 2021	SELECT-PSA 1	56	bDMARD-naïve	100	82.3	51.6±12.2	55.5	429	Upadacitinib 15 mg qd oral
McInnes, et al, 2021	McInnes, et al, 2021	SELECT-PSA 1	56	bDMARD-naïve	100	81.8	49.9±12.4	55.8	423	Upadacitinib 30 mg qd oral
McInnes, et al, 2021	McInnes, et al, 2021	SELECT-PSA 1	56	bDMARD-naïve	100	80.9	51.4±12.0	51.7	429	Adalimumab 40 mg q2w s.c.
Mease, et al, 2021	Mease, et al, 2021	SELECT-PsA 2	56	bDMARD-IR	0	43.6	53.0±12.0	53.6	211	Upadacitinib 15 mg qd oral
Mease, et al, 2021	Mease, et al, 2021	SELECT-PsA 2	56	bDMARD-IR	0	42.7	53.0±11.9	52.8	218	Upadacitinib 30 mg qd oral
Gottlieb, et al, 2009			36	Mixed	76	20	50	41	76	Ustekinumab 63 or 90 mg q4w s.c.
McInnes, et al, 2013	Kavanaugh, et al, 2015	PSUMMIT 1	108	bDMARD-naïve	100	48.3	48	48.3	205	Ustekinumab 45 mg weeks 0,4 + q12w s.c.
McInnes, et al, 2013	Kavanaugh, et al, 2015	PSUMMIT 1	108	bDMARD-naïve	100	49.5	47	43.1	204	Ustekinumab 90 mg weeks 0,4 + q12w s.c.
Ritchlin,			60	bDMARD-IR	0	52.4	49	53.4	103	Ustekinumab

Study	Study last extension	Name of study	Duration total (weeks)	Population	bDMARD-naïve %	Concomitant csDMARD %	Age (mean)	Female (%)	Number of patients	Drug and doses
et al, 2014		PSUMMIT 2								45 mg weeks 0,4 + q12w s.c.
Ritchlin, et al, 2014		PSUMMIT 2	60	bDMARD-IR	0	49.5	48	53.3	105	Ustekinumab 90 mg weeks 0,4 + q12w s.c.
van den Bosch, et al, 2002			12	bDMARD-naïve	100	0	48 (median)	44.4	9	Infliximab 5 mg/kg weeks 0,2,6 IV
Mease, et al, 2020		AMVISION Pooled	24	Mixed	69.8		48.6±12.8	50.3	318	Brodalumab 140 mg weeks 0,1 + q2w s.c.
Mease, et al, 2020		AMVISION Pooled	24	Mixed	68.3		48.1±12.4	47.8	321	Brodalumab 210 mg weeks 0,1 + q2w s.c.

Table S3 Rare causes of reported opportunistic infections

**Opportunistic infections**		
5 oral fungal infections adjudicated as OIs	Upadacitinib, 15 mg qd oral (1 patient)	Upadacitinib, 30 mg qd oral (4 patients)
3 *Pneumocystis jirovecii* infections	Abatacept, 125mg s.c (1 patient)	Upadacitinib, 30 mg qd oral (2 patients)
3 esophageal fungal infections adjudicated as OIs	Guselkumab, 100mg s.c. q4w	
2 OIs of unspecified cause	Bimekizumab, 160mg s.c. q4w (1 patient)	Bimekizumab, 320mg s.c. q4w (1 patient)
2 cytomegalovirus infections	Upadacitinib, 30 mg qd oral	
1 *Listeria* meningitis	Guselkumab, 100mg s.c. q4w	
1 *Legionella* infection	Golimumab, 100mg s.c. q4w	
1 histoplasmosis	Golimumab, 100mg s.c. q4w	
1 herpes simplex virus serious infection	Adalimumab, 40mg s.c. q2w	
1 eye toxoplasmosis	Golimumab, 100mg s.c. q4w	
1 coccidiomycosis infection	Upadacitinib, 15 mg qd oral	
1 bronchopulmonary aspergillosis	Upadacitinib, 15 mg qd oral	

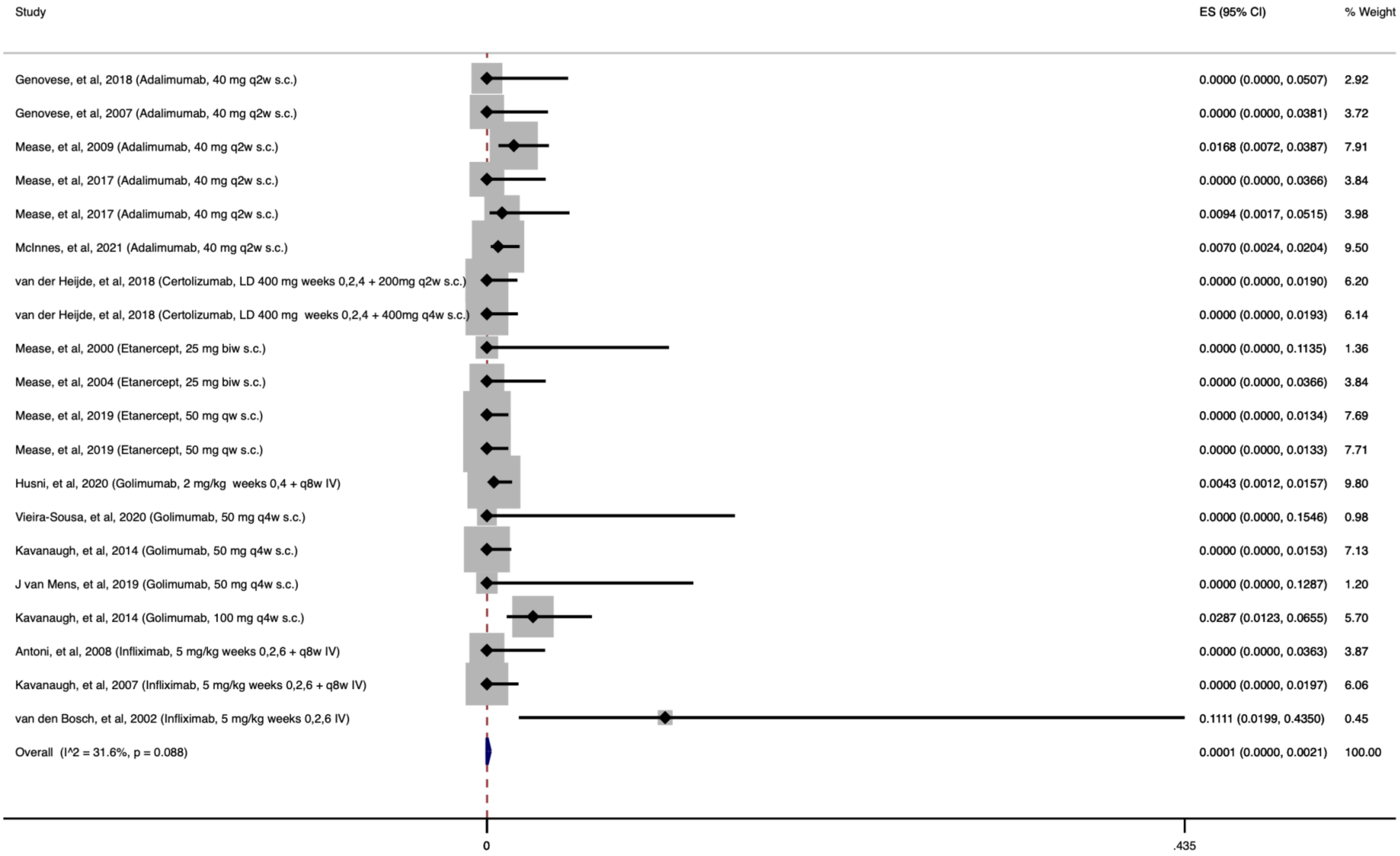

Figure S1 Opportunistic infections cumulative incidence for anti-TNFs during RCTs and their extension periods

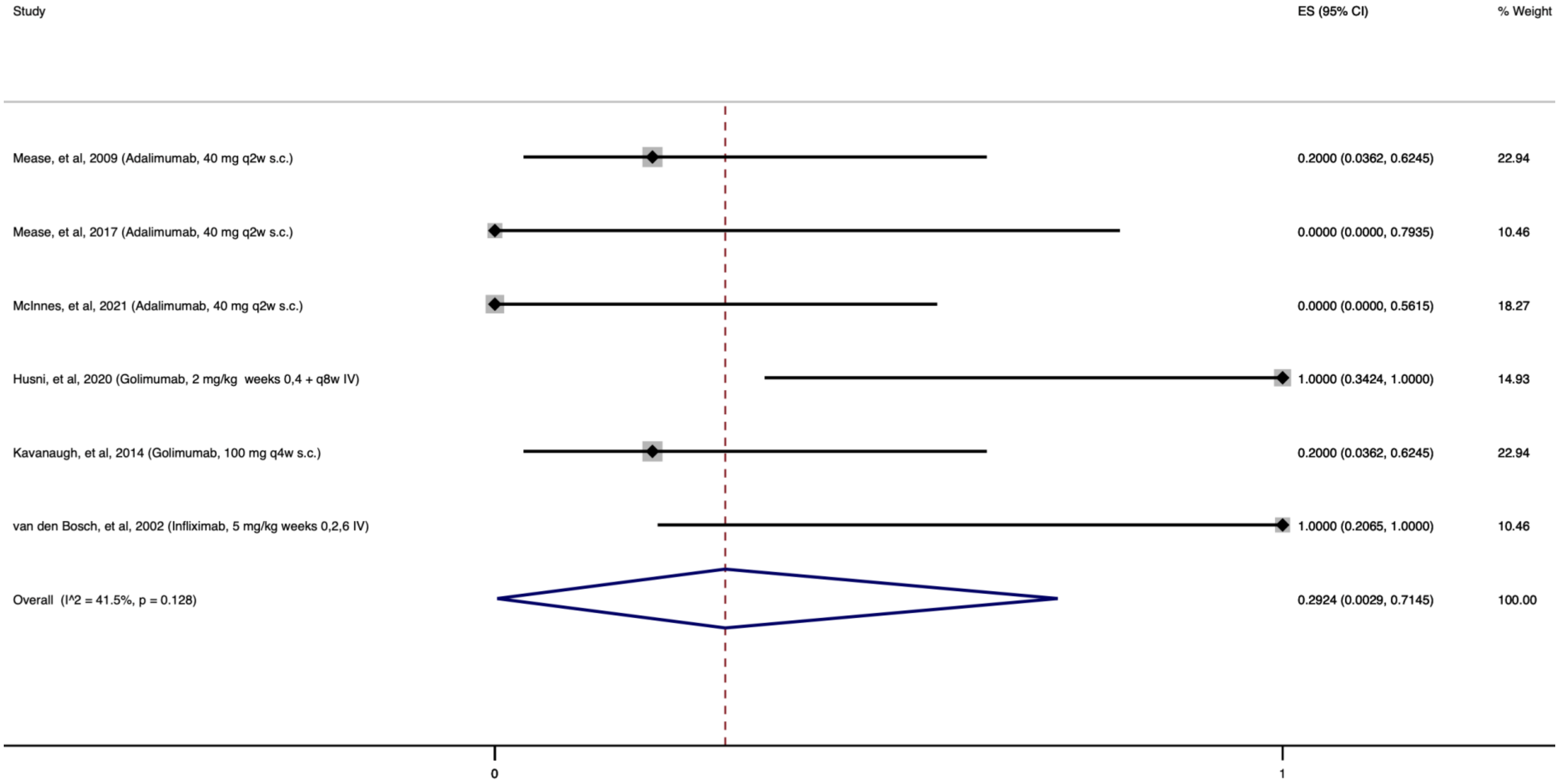

Figure S2 Pooled estimated proportion of *Mycobacterium tuberculosis* infections in patients with opportunistic infections receiving anti-TNFs

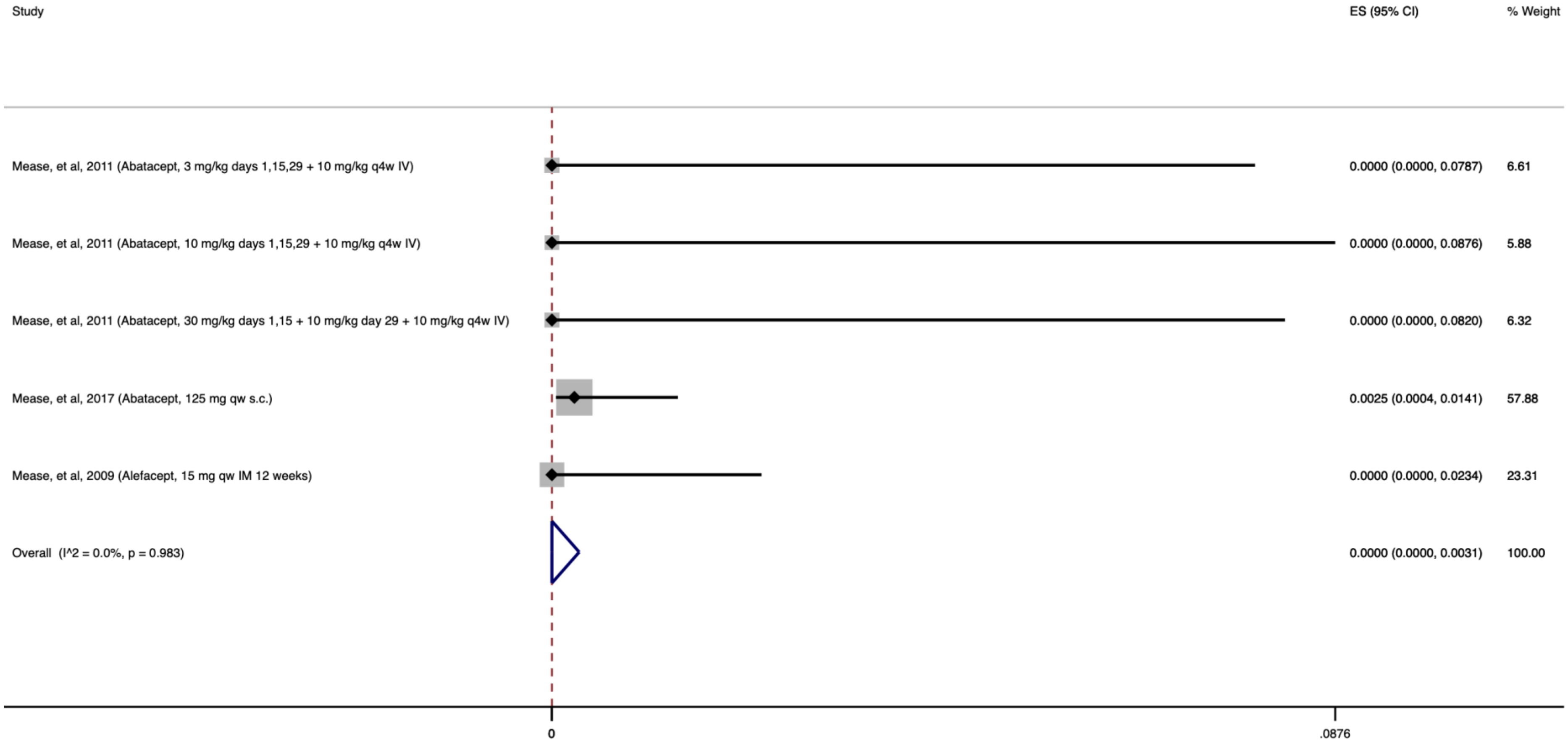

Figure S3 Opportunistic infections cumulative incidence for T-cell costimulation modulators during RCTs and their extension periods

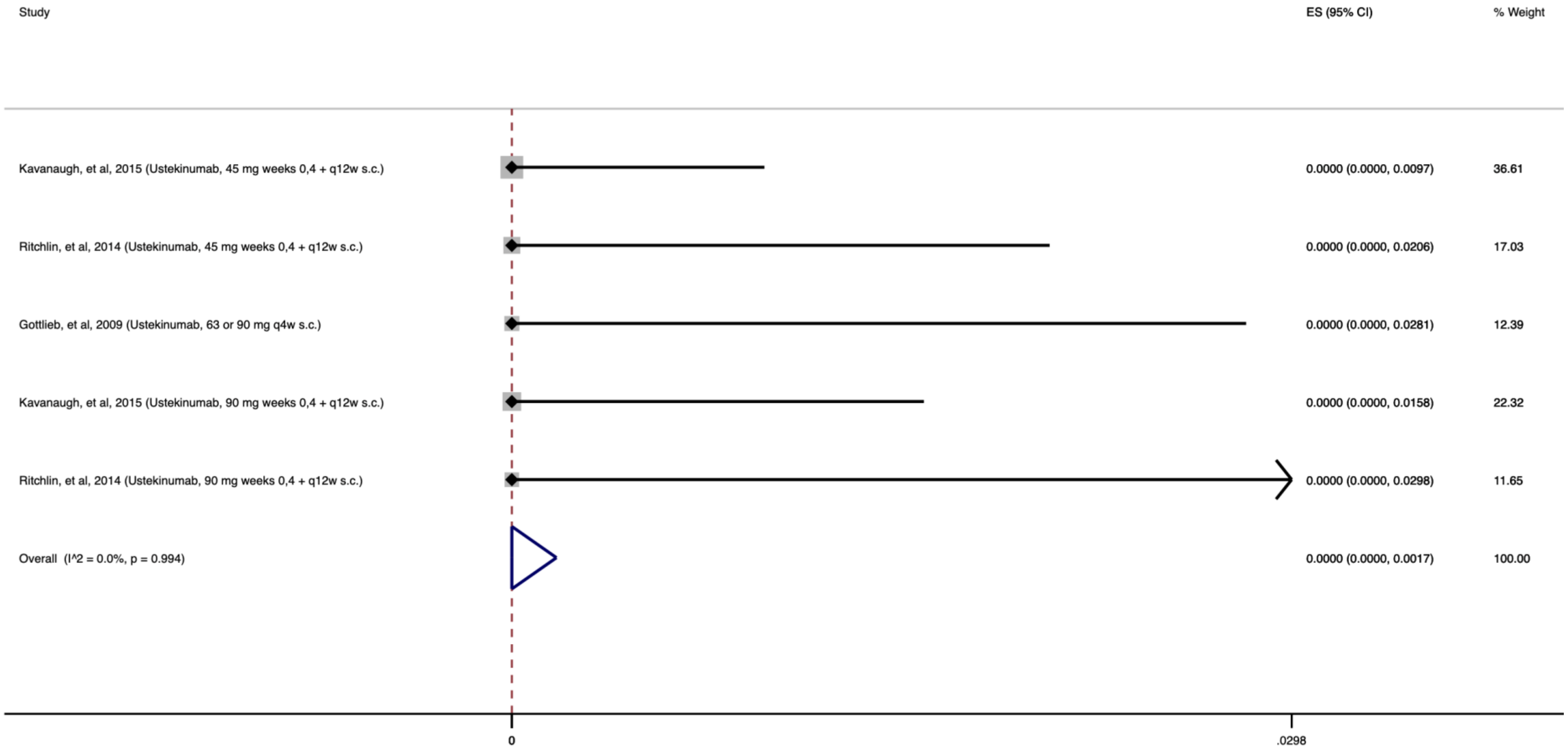

Figure S4 Opportunistic infections cumulative incidence for anti-IL-12/23 during RCTs and their extension periods

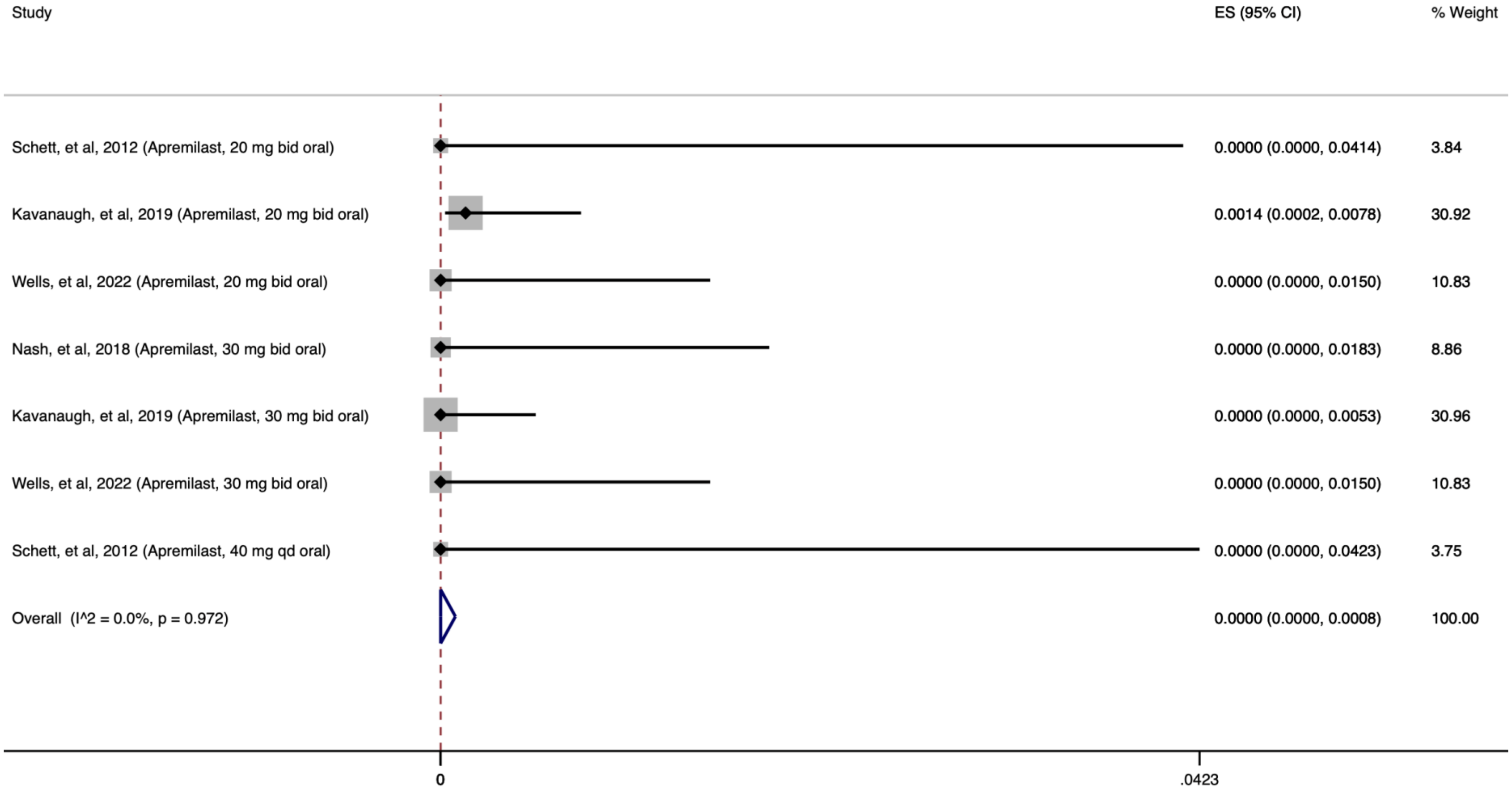

Figure S5 Opportunistic infections cumulative incidence for PDE4 inhibitors during RCTs and their extension periods

Study	Treatment Yes	Treatment No	Control Yes	Control No	rr with 95% CI	Weight (%)
Mease, et al, 2018 (Adalimumab, 40 mg q2w s.c.)	0	72	0	24	0.34 [0.01, 16.81]	4.71
Genovese, et al, 2007 (Adalimumab, 40 mg q2w s.c.)	0	51	0	49	0.96 [0.02, 47.53]	4.70
Mease, et al, 2005 (Adalimumab, 40 mg q2w s.c.)	0	151	0	162	1.07 [0.02, 53.71]	4.66
Mease, et al, 2014 (Certolizumab, LD 400 mg weeks 0,2,4 + 200mg q2w s.c.)	0	138	0	136	0.99 [0.02, 49.32]	4.67
Mease, et al, 2014 (Certolizumab, LD 400 mg weeks 0,2,4 + 400mg q4w s.c.)	0	135	0	136	1.01 [0.02, 50.40]	4.67
Mease, et al, 2000 (Etanercept, 25 mg biw s.c.)	0	30	0	30	1.00 [0.02, 48.82]	4.73
Mease, et al, 2004 (Etanercept, 25 mg biw s.c.)	0	101	0	104	1.03 [0.02, 51.39]	4.67
Mease, et al, 2019 (Etanercept, 50 mg qw s.c.)	0	283	0	284	1.00 [0.02, 50.40]	4.66
Mease, et al, 2019 (Etanercept, 50 mg qw s.c.)	0	284	0	284	1.00 [0.02, 50.22]	4.66
Vieira-Sousa, et al, 2020 (Golimumab, 50 mg q4w s.c.)	0	21	0	23	1.09 [0.02, 52.67]	4.75
Kavanaugh, et al, 2009 (Golimumab, 50 mg q4w s.c.)	0	146	0	113	0.78 [0.02, 38.79]	4.67
Kavanaugh, et al, 2009 (Golimumab, 100 mg q4w s.c.)	0	146	0	113	0.78 [0.02, 38.79]	4.67
Kavanaugh, et al, 2017 (Golimumab, 2 mg/kg weeks 0,4 + q8w IV)	0	240	0	239	1.00 [0.02, 49.98]	4.66
J van Mens, et al, 2019 (Golimumab, 50 mg q4w s.c.)	0	26	0	25	0.96 [0.02, 46.76]	4.74
Antoni, et al, 2005 (Infliximab, 5 mg/kg weeks 0,2,6 + q8w IV)	0	52	0	52	1.00 [0.02, 49.47]	4.69
Antoni, et al, 2005 (Infliximab, 5 mg/kg weeks 0,2,6 + q8w IV)	0	100	0	100	1.00 [0.02, 49.91]	4.67
Mease, et al, 2017 (Adalimumab, 40 mg q2w s.c.)	0	101	0	106	1.05 [0.02, 52.37]	4.67
Mease, et al, 2017 (Adalimumab, 40 mg q2w s.c.)	0	106	0	105	0.99 [0.02, 49.47]	4.67
McInnes, et al, 2021 (Adalimumab, 40 mg q2w s.c.)	0	429	3	420	0.14 [0.01, 2.72]	8.15
van den Bosch, et al, 2002 (Infliximab, 5 mg/kg weeks 0,2,6 IV)	1	8	0	9	3.00 [0.14, 65.16]	7.54
**Overall**					0.87 [0.37, 2.01]	

Heterogeneity: $\tau^2 = 0.00$, $I^2 = 0.00\%$, $H^2 = 1.00$

Test of $\theta_i = \theta_j$: $Q(19) = 2.39$, $p = 1.00$

Test of $\theta = 0$: $z = -0.34$, $p = 0.74$

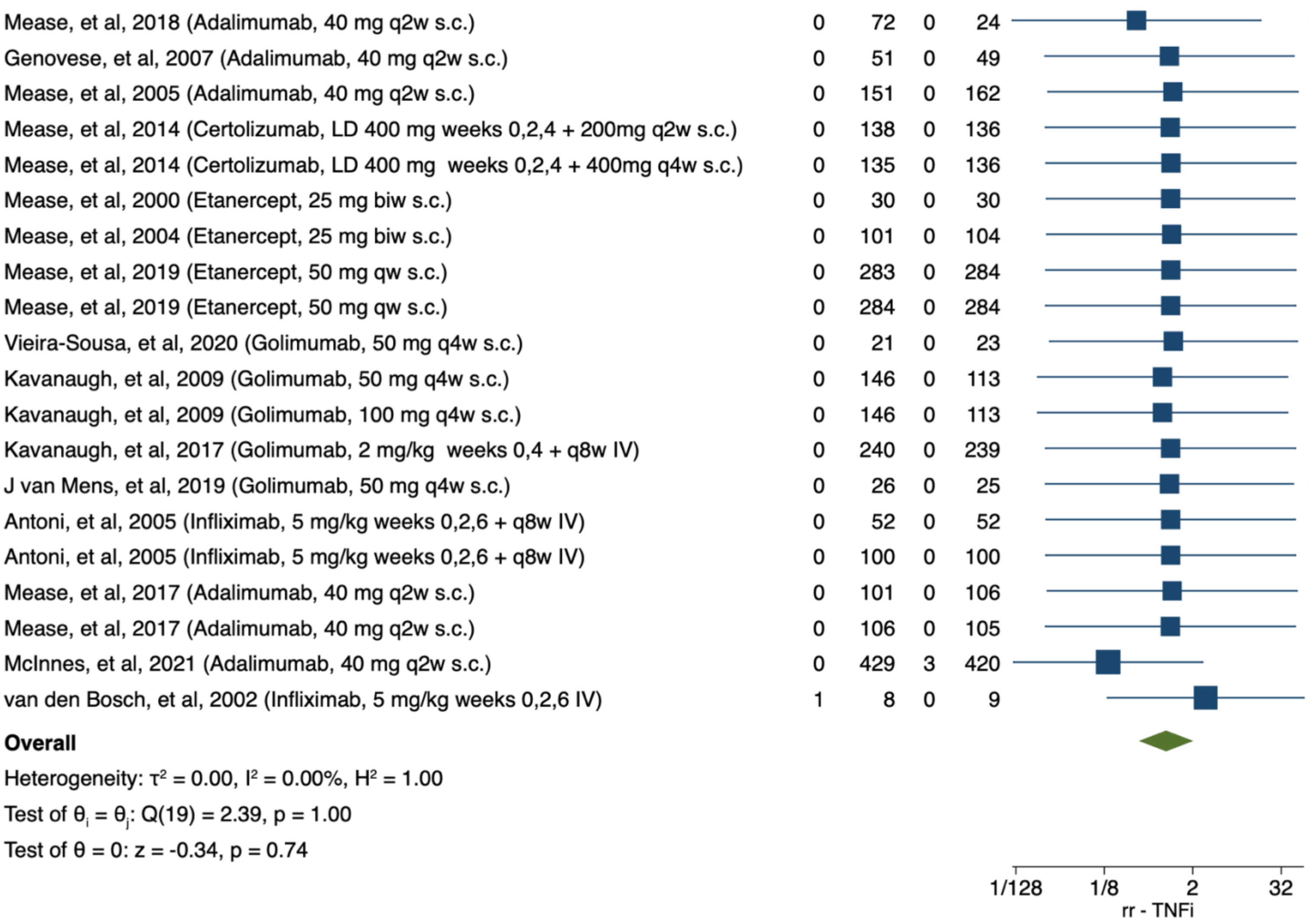

Random-effects REML model

Figure S6 Relative risk of anti-TNFs for opportunistic infections compared to placebo during placebo controlled period

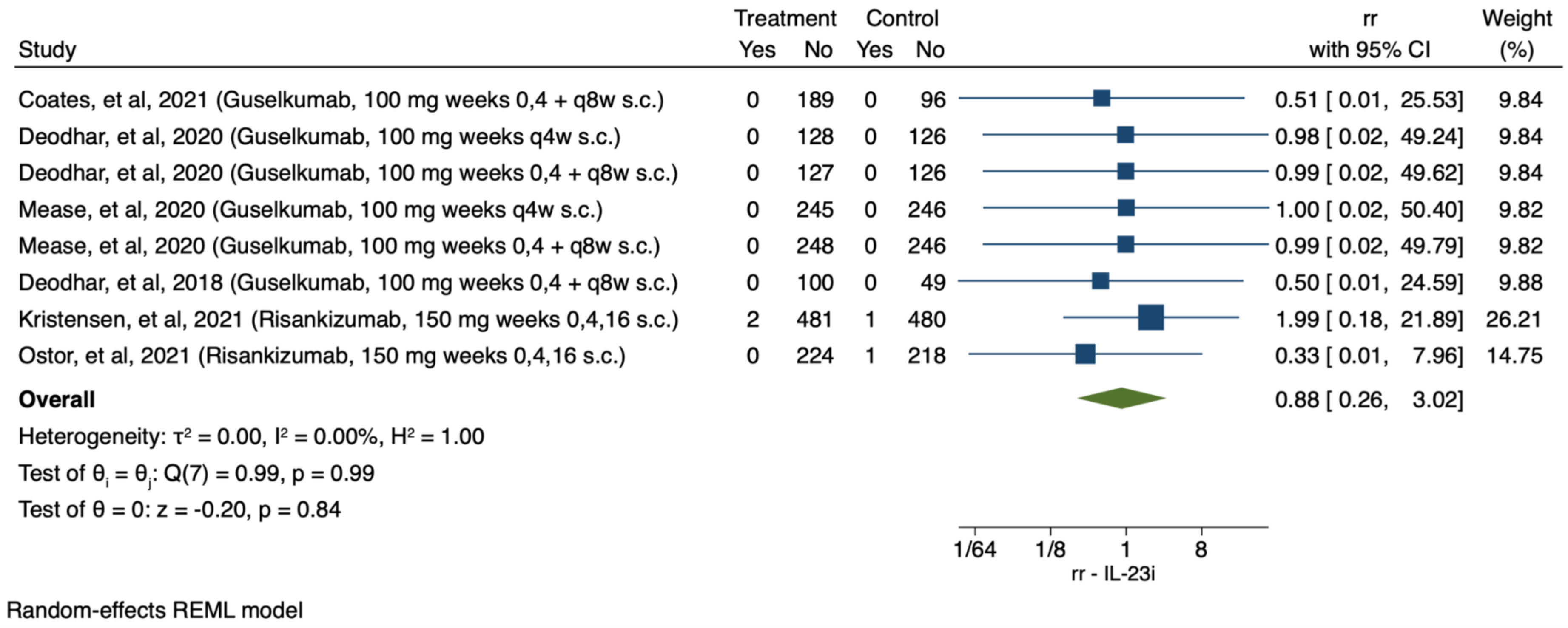

Figure S7 Relative risk of anti-IL-23 for opportunistic infections compared to placebo during placebo controlled period

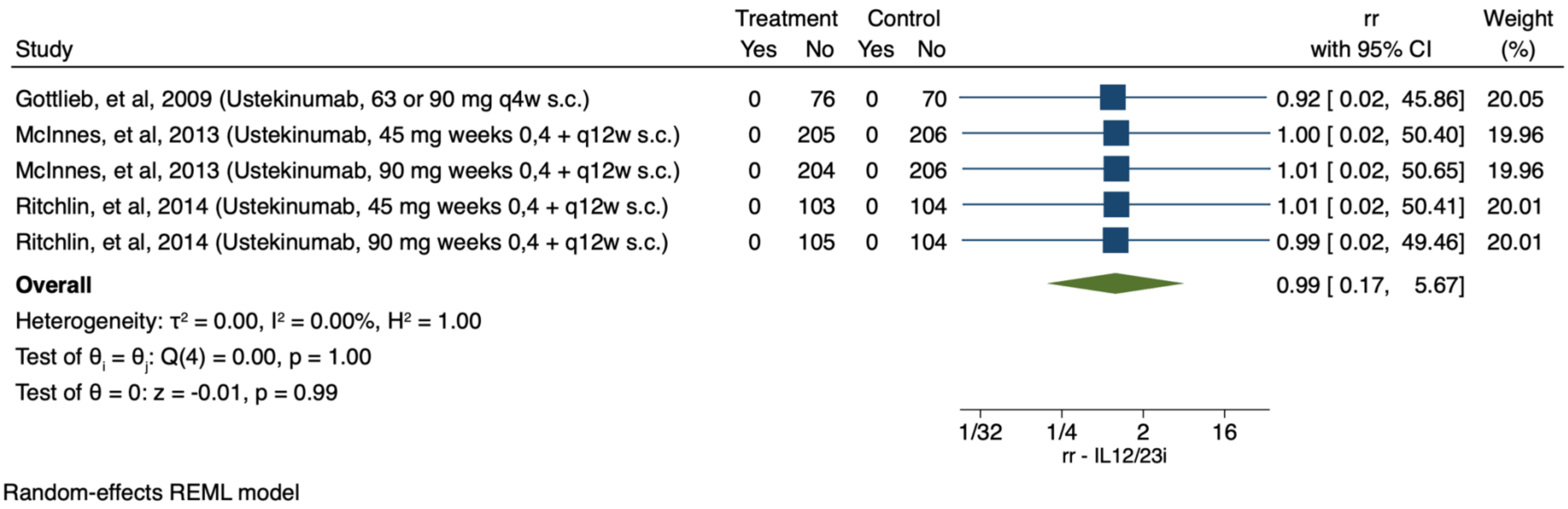

Figure S8 Relative risk of anti-IL-12/23 for opportunistic infections compared to placebo during placebo controlled period

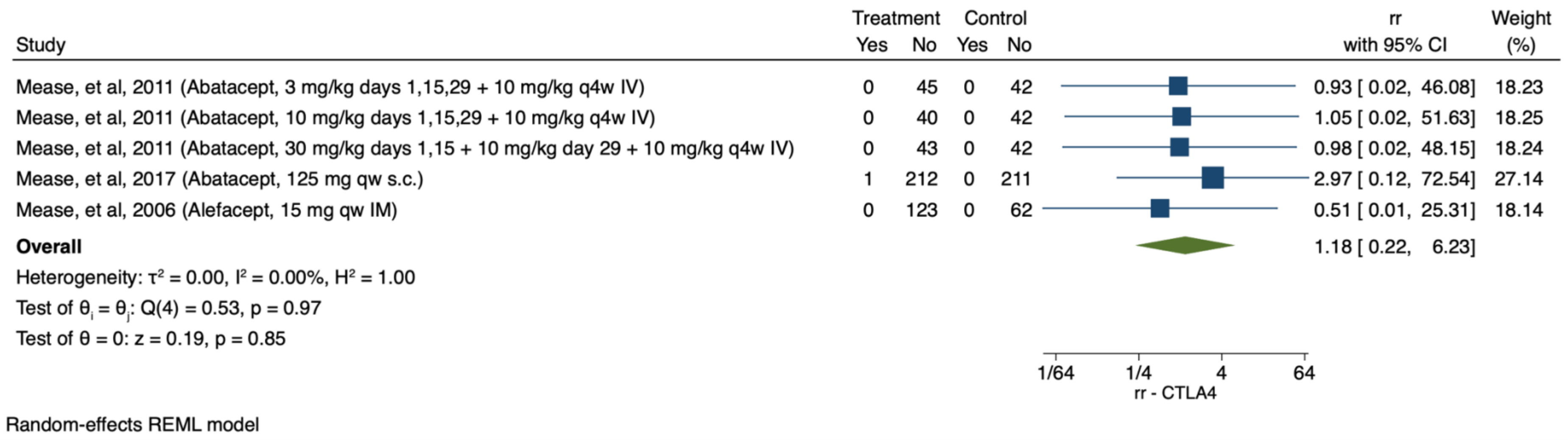

Figure S9 Relative risk of T-cell co-stimulation modulators for opportunistic infections compared to placebo during placebo controlled period

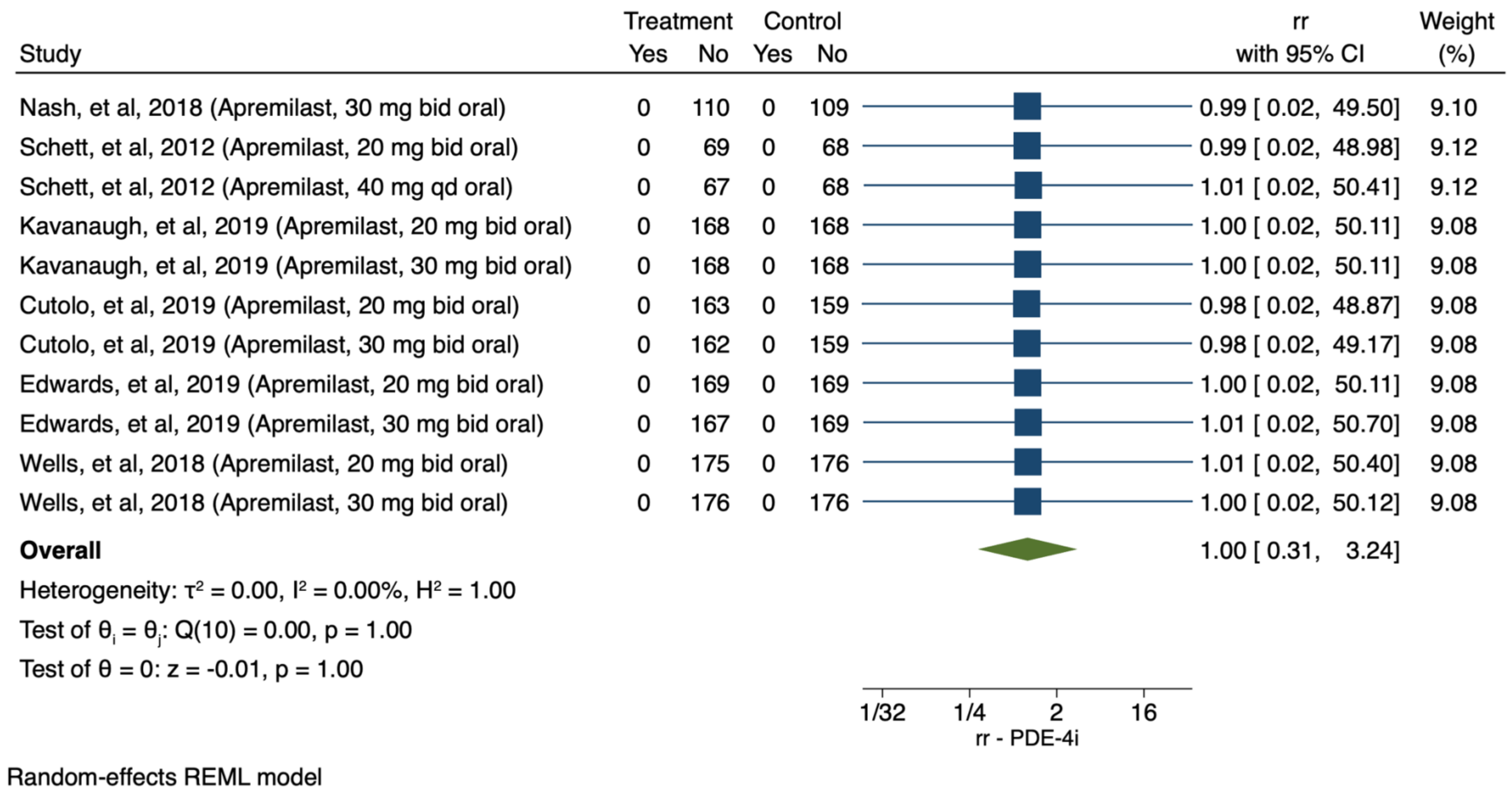

Figure S10 Relative risk of PDE4 inhibitors for opportunistic infections compared to placebo during placebo controlled period

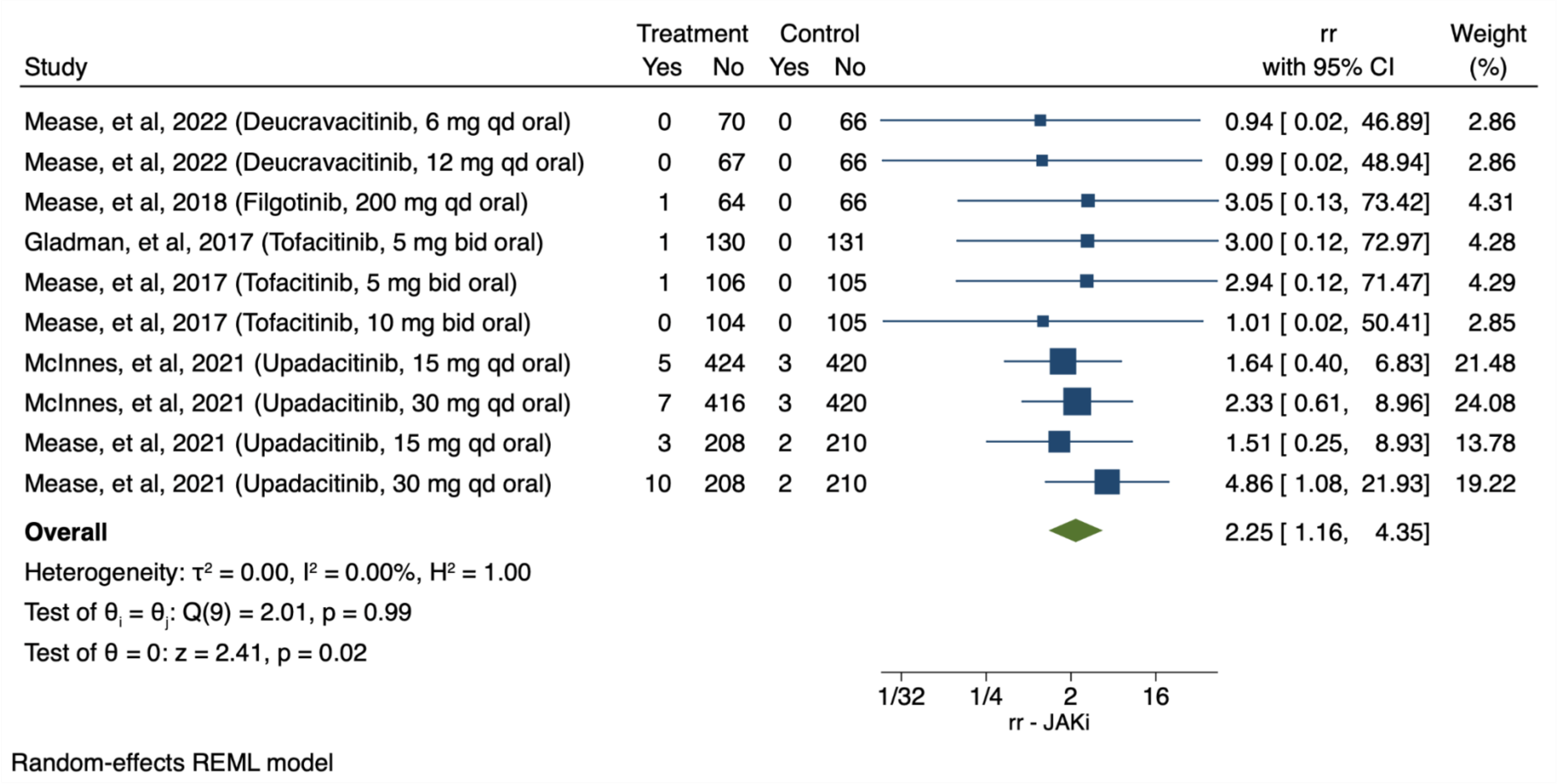

Figure S11 Relative risk of JAK inhibitors for opportunistic infections compared to placebo during placebo controlled period

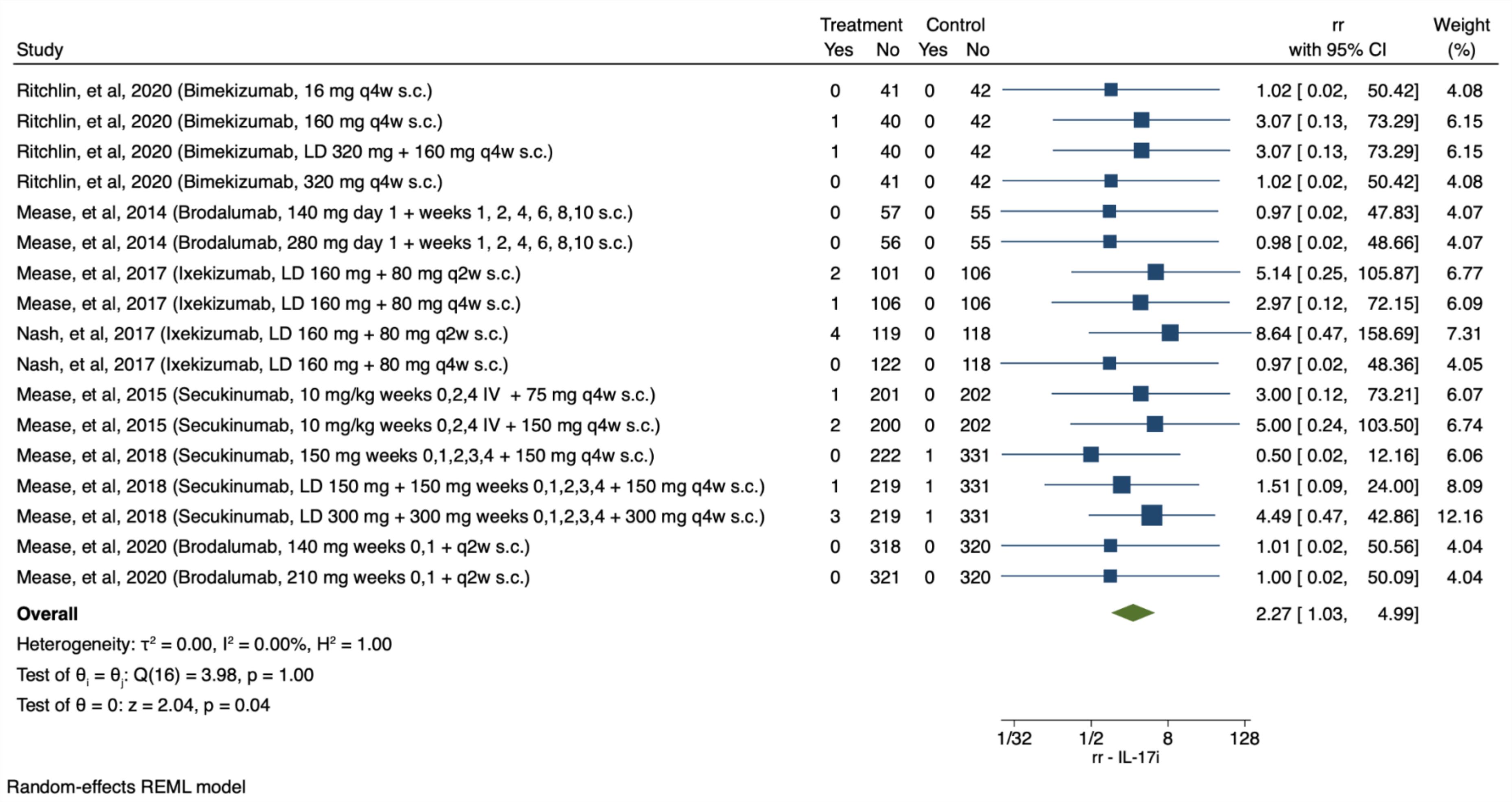

Figure S12 Relative risk of anti-IL-17 for opportunistic infections compared to placebo during placebo controlled period

Study	Study extension	Name of study	D1 Algorithm	D1 Assessment	D2 Algorithm	D2 Assessment	D3 Algorithm	D3 Assessment	D4 Algorithm	D4 Assessment	D5 Algorithm	D5 Assessment	Overall Algorithm
Mease, et al, 2011			Low	Low	Low	Low	Low	Low	Low	Low	Low	Low	Low
Mease, et al, 2017		ASTRAEA	Low	Low	Low	Low	Low	Low	Some concerns	Low	Low	Low	Low
Mease, et al, 2018	Genovese, et al, 2018		Low	Low	Low	Low	Low	Low	Some concerns	Low	Low	Low	Low
Genovese, et al, 2007			Low	Low	Low	Low	Low	Low	Some concerns	Low	Low	Low	Low
Mease, et al, 2005	Mease, et al, 2009	ADEPT	Low	Low	Low	Low	Low	Low	Some concerns	Low	Low	Low	Low
Mease, et al, 2006	Mease, et al, 2009		Low	Low	Low	Low	Low	Low	Some concerns	Low	Low	Low	Low
Nash, et al, 2018		ACTIVE	Low	Low	Low	Low	Low	Low	Some concerns	Low	Low	Low	Low
Schett, et al, 2012			Low	Low	Low	Low	Low	Low	Some concerns	Low	Low	Low	Low
Kavanaugh, et al, 2014	Kavanaugh, et al, 2019	PALACE 1	Low	Low	Low	Low	Low	Low	Some concerns	Low	Low	Low	Low
Cutolo, et al, 2016	Kavanaugh, et al, 2019	PALACE 2	Low	Low	Low	Low	Low	Low	Some concerns	Low	Low	Low	Low
Edwards et, al 2016	Kavanaugh, et al, 2019	PALACE 3	Low	Low	Low	Low	Low	Low	Some concerns	Low	Low	Low	Low
Wells, et al, 2018	Wells, et al, 2022	PALACE 4	Low	Low	Low	Low	Low	Low	Low	Low	Low	Low	Low
Ritchlin, et al, 2020		BE ACTIVE	Low	Low	Low	Low	Low	Low	Some concerns	Low	Low	Low	Low
Mease, et al, 2014			Low	Low	Low	Low	Low	Low	Some concerns	Low	Low	Low	Low
Mease, et al, 2014	van der Heijde, et al, 2018	RAPID-PsA	Low	Low	Low	Low	Low	Low	Some concerns	Low	Low	Low	Low
Mease, et al, 2022			Low	Low	Low	Low	Low	Low	Low	Low	Low	Low	Low
Mease, et al, 2000			Low	Low	Low	Low	Low	Low	Low	Low	Low	Low	Low
Mease, et al, 2004			Low	Low	Low	Low	Low	Low	Low	Low	Low	Low	Low
Mease, et al, 2019		SEAM-PsA	Low	Low	Low	Low	Low	Low	Low	Low	Low	Low	Low
Mease, et al, 2018		EQUATOR	Low	Low	Low	Low	Low	Low	Low	Low	Low	Low	Low
Vieira-Sousa, et al, 2020		GO-DACT	Low	Low	Low	Low	Low	Low	Low	Low	Low	Low	Low
Kavanaugh, et al, 2009	Kavanaugh, et al, 2014	GO-REVEAL	Low	Low	Low	Low	Low	Low	Some concerns	Low	Low	Low	Low
Kavanaugh, et al, 2017	Husni, et al, 2020	GO-VIBRANT	Low	Low	Low	Low	Low	Low	Some concerns	Low	Low	Low	Low
J van Mens, et al, 2019			Low	Low	Low	Low	Low	Low	Some concerns	Low	Low	Low	Low
Coates, et al, 2021	Coates, et al, 2021	COSMOS	Low	Low	Low	Low	Low	Low	Some concerns	Low	Low	Low	Low
Deodhar, et al, 2020	Ritchlin, et al, 2021	DISCOVER-1	Low	Low	Low	Low	Low	Low	Some concerns	Low	Low	Low	Low
Mease, et al, 2020	McInnes, et al, 2022	DISCOVER-2	Low	Low	Low	Low	Low	Low	Some concerns	Low	Low	Low	Low
Deodhar, et al, 2018			Low	Low	Low	Low	Low	Low	Some concerns	Low	Low	Low	Low
Antoni, et al, 2005	Antoni, et al, 2008	IMPACT	Low	Low	Low	Low	Low	Low	Some concerns	Low	Low	Low	Low
Antoni, et al, 2005	Kavanaugh, et al, 2007	IMPACT 2	Low	Low	Low	Low	Low	Low	Some concerns	Low	Low	Low	Low
Mease, et al, 2017		SPIRIT-P1	Low	Low	Low	Low	Low	Low	Low	Low	Low	Low	Low
Nash, et al, 2017	Orbai, et al, 2021	SPIRIT-P2	Low	Low	Low	Low	Low	Low	Some concerns	Low	Low	Low	Low
Kristensen, et al, 2021		KEEPsAKE 1	Low	Low	Low	Low	Low	Low	Low	Low	Low	Low	Low
Ostor, et al, 2021		KEEPsAKE 2	Low	Low	Low	Low	Low	Low	Low	Low	Low	Low	Low
Mease, et al, 2015	Kavanaugh, et al, 2017	FUTURE 1	Low	Low	Low	Low	Low	Low	Some concerns	Low	Low	Low	Low
McInnes, et al, 2015		FUTURE 2	Low	Low	Low	Low	Low	Low	Some concerns	Low	Low	Low	Low
Nash, et al, 2018		FUTURE 3	Low	Low	Low	Low	Low	Low	Some concerns	Low	Low	Low	Low
Mease, et al, 2018	Mease, et al, 2021	FUTURE 5	Low	Low	Low	Low	Low	Low	Some concerns	Low	Low	Low	Low
Gladman, et al, 2017		OPAL Beyond	Low	Low	Low	Low	Low	Low	Some concerns	Low	Low	Low	Low
Mease, et al, 2017		OPAL Broaden	Low	Low	Low	Low	Low	Low	Some concerns	Low	Low	Low	Low
McInnes, et al, 2021	McInnes, et al, 2021	SELECT-PSA 1	Low	Low	Low	Low	Low	Low	Some concerns	Low	Low	Low	Low
Mease, et al, 2021	Mease, et al, 2021	SELECT-PsA 2	Low	Low	Low	Low	Low	Low	Some concerns	Low	Low	Low	Low
Gottlieb, et al, 2009			Low	Low	Low	Low	Low	Low	Some concerns	Low	Low	Low	Low
McInnes, et al, 2013	Kavanaugh, et al, 2015	PSUMMIT 1	Low	Low	Low	Low	Low	Low	Some concerns	Low	Low	Low	Low
Ritchlin, et al, 2014		PSUMMIT 2	Low	Low	Low	Low	Low	Low	Some concerns	Low	Low	Low	Low
van den Bosch, et al, 2002			Low	Low	Low	Low	Low	Low	Low	Low	Low	Low	Low
Mease, et al, 2020		AMVISION Pooled	Low	Low	Low	Low	Low	Low	Low	Low	Low	Low	Low

Figure S13 Individual study risk of bias in each domain and overall
